# Increasing hub disruption parallels dementia severity in autosomal dominant Alzheimer’s disease

**DOI:** 10.1162/netn_a_00395

**Published:** 2024-12-10

**Authors:** Jiaxin Cindy Tu, Peter R. Millar, Jeremy F. Strain, Andrew Eck, Babatunde Adeyemo, Abraham Z. Snyder, Alisha Daniels, Celeste Karch, Edward D. Huey, Eric McDade, Gregory S. Day, Igor Yakushev, Jason Hassenstab, John Morris, Jorge J. Llibre-Guerra, Laura Ibanez, Mathias Jucker, Patricio Chrem Mendez, Richard J. Perrin, Tammie L. S. Benzinger, Clifford R. Jack, Richard Betzel, Beau M. Ances, Adam T. Eggebrecht, Brian A. Gordon, Muriah D. Wheelock, the Dominantly Inherited Alzheimer Network

**Affiliations:** Department of Radiology, Washington University in St. Louis, St. Louis, MO, USA; Department of Neurology, Washington University in St. Louis, St. Louis, MO, USA; Department of Psychiatry, Washington University in St. Louis, St. Louis, MO, USA; Department of Psychiatry and Human Behavior, Warren Alpert Medical School of Brown University, Providence, RI, USA; Department of Neurology, Mayo Clinic, Jacksonville, FL, USA; Department of Nuclear Medicine, Technical University of Munich, Munich, Germany; NeuroGenomics and Informatics Center, Washington University in St. Louis, St. Louis, MO, USA; Department of Cellular Neurology, Hertie Institute for Clinical Brain Research, University of Tübingen, Tübingen, Germany; German Center for Neurodegenerative Diseases (DZNE), Tübingen, Germany; Memory Center, Fleni, Argentina; Department of Pathology and Immunology, Washington University in St. Louis, St. Louis, MO, USA; Department of Radiology, Mayo Clinic, Rochester, MN, USA; Department of Psychological and Brain Sciences, Indiana University, Bloomington, IN, USA; A full list of members appears in the Supporting Information.

**Keywords:** Neurodegeneration, Functional connectivity, Hubs, Alzheimer’s disease, Biomarker

## Abstract

Hub regions in the brain, recognized for their roles in ensuring efficient information transfer, are vulnerable to pathological alterations in neurodegenerative conditions, including Alzheimer’s disease (AD). Computational simulations and animal experiments have hinted at the theory of activity-dependent degeneration as the cause of this hub vulnerability. However, two critical issues remain unresolved. First, past research has not clearly distinguished between two scenarios: hub regions facing a higher risk of connectivity disruption (targeted attack) and all regions having an equal risk (random attack). Second, human studies offering support for activity-dependent explanations remain scarce. We refined the hub disruption index to demonstrate a hub disruption pattern in functional connectivity in autosomal dominant AD that aligned with targeted attacks. This hub disruption is detectable even in preclinical stages, 12 years before the expected symptom onset and is amplified alongside symptomatic progression. Moreover, hub disruption was primarily tied to regional differences in global connectivity and sequentially followed changes observed in amyloid-beta positron emission tomography cortical markers, consistent with the activity-dependent degeneration explanation. Taken together, our findings deepen the understanding of brain network organization in neurodegenerative diseases and could be instrumental in refining diagnostic and targeted therapeutic strategies for AD in the future.

## Author Summary

Our research introduces a refined hub disruption index that reveals early and progressive targeted connectivity impairments in brain regions central to information transfer in Alzheimer’s disease (AD). Detectable up to 12 years before clinical symptoms, selective functional connectivity impairments were higher at high global connectivity regions, preceding changes in amyloid-beta positron emission tomography markers. This supports the concept of activity-dependent degeneration and underscores the vulnerability of hub regions to neurodegenerative processes. Our findings enhance the understanding of the brain’s network organization in AD and offer significant potential for improving early diagnosis and developing precise therapeutic interventions.

## INTRODUCTION

Alzheimer’s disease (AD) is characterized by a cascade of complex pathological changes in the brain including amyloid-beta (A*β*) aggregation and the formation of neurofibrillary tangles composed of the tau protein, resulting in neurodegeneration (Musiek & Holtzman, 2015). While these microscopic changes have been well-documented, there is a growing interest in understanding how these pathologies translate to altered brain connectivity observed in AD patients. Resting-state functional connectivity (FC), measured by temporal correlations of the blood oxygen level-dependent signals between regions of the brain from fMRI data collected in a task-free state (Biswal et al., 1995), differs in cognitively impaired and preclinical AD individuals versus cognitively normal controls (Chhatwal et al., 2013; Dennis & Thompson, 2014; Sheline & Raichle, 2013; Smith et al., 2021; Yu et al., 2021). FC is a potential imaging marker for AD (Buckley et al., 2017; Dickerson & Sperling, 2005; Sorg et al., 2009; Thomas et al., 2014) since disruptions in connectivity between brain regions may be linked to synaptic changes before cell death and atrophy (Drzezga et al., 2011).

Prior imaging studies have suggested that the posterior parts of the default mode network (DMN) deteriorate earlier than the anterior parts in AD, providing evidence for a cascading network failure mechanism (Jones et al., 2016, 2017). Importantly, the initiation of amyloid (Gordon et al., 2018; Palmqvist et al., 2017) and tau (Frontzkowski et al., 2022) pathologies, as well as the rate of their accumulation (Liu et al., 2023; Villain et al., 2012), in the brain is not spatially homogenous, providing converging evidence for differences in regional vulnerability to pathological changes. However, precisely what underlies the sequence of regional FC failure, as well as how FC disruptions relate to the molecular pathology, is unknown.

Hub regions (van den Heuvel & Sporns, 2013) are highly connected nodes with high network centrality that play a critical role in facilitating efficient communication and integration of information across different regions of complex networks. Brain hubs are affected across multiple diseases (Crossley et al., 2014), including AD (Dai et al., 2015; Yu et al., 2017). One hypothesis for the cause of hub vulnerability to pathology and degeneration is that hub regions are selectively targeted by activity-dependent damage (de Haan et al., 2012). Several lines of evidence support this hypothesis: (a) Hubs have high metabolic demands (Bullmore & Sporns, 2012; Drzezga et al., 2011; Tomasi et al., 2013; Vaishnavi et al., 2010); (b) they are especially susceptible to A*β* deposition in AD (Buckner et al., 2009; Bullmore & Sporns, 2012; Drzezga et al., 2011; Myers et al., 2014); and (c) they serve as the spreading centers for tau pathology (Cope et al., 2018; Franzmeier et al., 2020; Frontzkowski et al., 2022). Due to their topologically central role, targeted attacks on hubs have a more deleterious effect on network efficiency (Achard et al., 2006; Alstott et al., 2009; Crossley et al., 2014; Honey & Sporns, 2008). Indeed, previous research has identified FC alterations particularly at hubs in AD patients (Drzezga et al., 2011; Stam et al., 2009; Yu et al., 2017), as well as in mice with extracellular amyloidosis (TgCRND8 mice) (Kotlarz et al., 2022). In vivo studies in mice also validated the relationship between neuronal activity level and A*β* deposition (Bero et al., 2011; Cirrito et al., 2005), suggesting that increasing amyloid burden through increased reference activity triggers hub disruptions. While there is a strong theoretical and empirical evidence to support the role of hub disruption in AD, little is known concerning the relationship between hub disruption, dementia severity, and symptomatic onset.

Here, we leveraged a unique population with autosomal dominant AD (ADAD), which allowed for accurate estimation of years to symptom onset (EYO) due to the highly predictable onset of cognitive decline (Bateman et al., 2012). With a highly penetrant genetic form of the disease, ADAD participants also have a high certainty in their AD dementia diagnosis (as opposed to sporadic AD). Furthermore, since ADAD-associated dysfunction generally occurs in a younger population (<60 years old) that experiences fewer AD-independent, aging-related neuropathology than is commonly seen in association with late-onset sporadic AD, we can test the associations of FC with AD pathology with fewer confounding aging-related co-pathology and estimate FC network characteristics with minimal age-related changes in neurovascular coupling (Fabiani et al., 2014).

We examined the regional vulnerability in terms of lower FC by measuring the FC hub disruption as a function of ADAD dementia progression from preclinical (Clinical Dementia Rating [CDR] = 0) to mild (CDR = 0.5, also known as mild cognitive impairment [MCI]), moderate, and severe dementia (CDR ≥ 1). We hypothesized that hub disruption is an early-emerging phenomenon that intensifies with disease progression. Finally, we investigated the relative timing of hub disruption compared with cortical amyloid deposition and cognitive decline. Our goals were to test the targeted attack on the hubs model in ADAD throughout disease progression and to obtain evidence for activity-dependent degeneration.

## MATERIALS AND METHODS

### Participants

Individuals were recruited from the Dominantly Inherited Alzheimer Network (DIAN) Observational Study (https://dian.wustl.edu/). Here, we examine the cross-sectional data from mutation carrier (MC; *N* = 122), with alterations in presenilin 1 (PSEN1), presenilin 2 (PSEN2), or the amyloid precursor proteins (APP), and unaffected noncarrier (NC; *N* = 85) family members (Supporting Information Table S1) (Bateman et al., 2012). The age at symptom onset is relatively consistent within families and mutation types; this allows participants to be staged by their EYO (Bateman et al., 2012; McKay et al., 2023). Both MCs and NCs have an EYO value based on their familial pedigree, but only MCs are expected to develop ADAD. The study was reviewed and approved by the Institutional Review Board at Washington University in St. Louis, and written informed consent forms were obtained from participants or their legally authorized representatives in accordance with their local Institutional Review Board. The data are from the data freeze 15.

### CDR Stages

We further categorized the MC by dementia severity using the global CDR (Morris, 1993) into three groups: cognitively normal (CDR = 0), very mild dementia (CDR = 0.5), and mild-to-severe dementia (CDR ≥ 1). To control for the effect of aging, we age-matched the NCs for each MC group according to the following procedure. First, *Z*-scores were calculated for the age and EYO values separately using their mean and standard deviation across all participants, resulting in a vector of size 2 × 1 for each participant. The Euclidean distances between the vectors were calculated, and the closest MC for each NC participant was determined, defining an age-matched group of NCs for each MC group (Table 1).

**Table 1.** Sample characteristics (MC and NC matches)

	**Measure**	**Mutation carrier (*N* = 69)** ^**a**^	**Noncarrier match (*N* = 52)** ^**b**^	***χ*** ^**2**^	***df***	***p* value**
MC CDR = 0/NC match 1	Sex (M/F)	36/33	23/29	0.749	1	0.387
	Family mutation (PS1, PS2, APP) *n* (%)	48 (70%)	36 (69%)	0.381	2	0.827
		11 (16%)	10 (19%)			
		10 (14%)	6 (11%)			
	APOE *ϵ*4 carriers/noncarriers	17/52	14/38	0.081	1	0.776
	**Median [min, max]**			**Mann-Whitney *U***	***z***	***p* value**
	Age (years)	33.2 [18.0, 52.6]	34.4 [21.5, 52.8]	4,030	−0.935	0.350
	Education (years)	16 [9, 24]	16 [10, 21]	4,198	−0.06	0.956
	EYO	−15.1 [−36.0, −0.5]	−15.3 [−31.5, −0.8]	4,098	−0.579	0.563
	CCS^c^	−0.03 [−1.35, 1.64]	0.10 [−1.14, 1.14]	3,817	−1.570	0.116
	Average FD of retained frames	0.085 [0.035, 0.223]	0.074 [0.039, 0.174]	4,561	1.840	0.066
	Remaining minutes of the scan	4.7 [3.2, 6.3]	4.3 [3.2, 17.3]	4,415.5	1.079	0.281
	**Measure**	**Mutation carrier (*N* = 32)**	**Noncarrier match (*N* = 17)**	***χ*** ^**2**^	***df***	***p* value**
MC CDR = 0.5/NC match 2	Sex (M/F)	13/19	3/14	2.666	1	0.103
	Family mutation (PS1, PS2, APP) *n* (%)	25 (78%)	8 (47%)	5.049	2	0.080
		1 (3%)	2 (12%)			
		6 (19%)	7 (41%)			
	APOE *ϵ*4 carriers/noncarriers	9/23	3/14	0.659	1	0.417
	**Median [min, max]**			**Mann-Whitney *U***	***z***	***p* value**
	Age (years)	48.5 [30.1, 65.6]	49.7 [29.1, 62.6]	824	0.494	0.622
	Education (years)	13.5 [6, 19]	14 [9, 18]	733.5	−1.398	0.162
	EYO	1.7 [−14.2, 10.4]	1.7 [−14.5, 8.9]	837	0.767	0.443
	CCS^d^	−1.55 [–3.09, −0.11]	0.01 [−1.24, 1.53]	487	−5.148	<0.001***
	Average FD of retained frames	0.106 [0.052, 0.194]	0.091 [0.045, 0.176]	850	1.040	0.299
	Remaining minutes of the scan	6.6 [3.4, 10.1]	4.6 [4.1, 16.7]	855.5	1.156	0.248
	**Measure**	**Mutation carrier (*N* = 20)**	**Noncarrier match (*N* = 15)**	***χ*** ^**2**^	***df***	***p* value**
MC CDR **≥** 1/NC match 3	Sex (M/F)	9/11	7/8	0.010	1	0.922
	Family mutation (PS1, PS2, APP) *n* (%)	17 (85%)	12 (80%)	6.276	2	0.043*
		0 (0%)	3 (20%)			
		3 (15%)	0 (0%)			
	APOE ϵ4 carriers/noncarriers	5/15	5/10	0.292	1	0.589
	**Median [min, max]**			**Mann-Whitney *U***	***z***	***p* value**
	Age (years)	50.8 [35.8, 67.0]	55.4 [36.4, 69.5]	341	−0.617	0.538
	Education (years)	12 [8, 18]	14 [11, 26]	292.5	−2.270	0.023*
	EYO	4.3 [−1.8, 14.9]	4.9 [−3.3, 20.8]	372	0.383	0.702
	CCS^e^	−2.71 [−3.43, −2.15]	−0.05 [−0.64, 1.07]	105	−4.561	<0.001***
	Average FD of retained frames	0.126 [0.054, 0.205]	0.113 [0.045, 0.192]	380	0.650	0.516
	Remaining minutes of the scan	4.5 [3.4, 10.0]	4.8 [3.2, 11.4]	316	−1.451	0.147

^a^ Removed one participant due to non-other participants existing from the same site.

^b^ Removed one participant due to non-other participants existing from the same site.

^c^ Missing two participants.

^d^ Missing two participants.

^e^ Missing six participants.

Medians and Mann-Whitney test statistics reported (Shapiro-Wilk test of normality *p* < 0.001)

*df*, degrees of freedom; CDR, Clinical Dementia Rating; M, male; F, female; EYO, estimated years from expected symptom onset; CCS, cognitive composite score.

*p < 0.05, **p < 0.01, ***p < 0.001.

### MRI Data Acquisition

Neuroimaging protocols have been previously published (McKay et al., 2023). Briefly, T1-weighted magnetization-prepared rapid acquisition gradient echo (MP-RAGE) images were acquired at multiple sites on Siemens 3T scanners (Erlangen, Germany). Resting-state fMRI scans were acquired with echo planar imaging (EPI) while participants were instructed to maintain visual fixation on a crosshair. The sequence details are provided in Supporting Information Table S2. “Pre-scan normalize” was enabled to minimize gain field inhomogeneities attributable to proximity to the receiver coils. Acquisition lasted ~6 min each run, and the number of acquired runs in the DIAN cohort varied between one and three.

### MRI Data Preprocessing

Details on preprocessing followed previously described methods (Smith et al., 2021; Strain et al., 2022) using the 4dfp suite of tools (https://4dfp.readthedocs.io). Briefly, slice timing correction and intensity normalization were performed. Head motion was corrected within and across runs. The initial atlas transformation was computed using affine registration of the fMRI data to an atlas-representative template via the MP-RAGE (EPI_mean_ → MP-RAGE → template). A final atlas transformation was performed after denoising. Frames with high motion, as measured by DVARS (frame-to-frame BOLD signal change over the entire brain) and the framewise displacement (FD) (Power et al., 2014), were censored. Due to the empirical observation that baseline DVARS in the absence of motion differs across individuals, the DVARS criterion was set individually as 2.5 standard deviations above the mean (see the Supporting Information in White III et al., 2020). Additionally, an FD (L2-norm of the six motion parameters) criterion of 0.4 mm was applied to further mitigate the effect of motion. Frames were censored if either criterion was met. We validated that our denoising strategy successfully mitigated the distance-dependent correlation with the remaining motion (measured as the mean FD of the remaining frames) (Supporting Information Figure S1). The time series were band-pass filtered between 0.005 and 0.1 Hz. Censored frames were approximated by linear interpolation for band-pass filtering only and were excluded from subsequent steps.

Denoising was then performed with a CompCor-like strategy (Behzadi et al., 2007). As previously described (Raut et al., 2019), nuisance regressors were derived from three compartments (white matter, ventricles, and extra-axial space) and were then dimensionality-reduced. White matter and ventricular masks were segmented in each participant using FreeSurfer 5.3 (Fischl, 2012) and were spatially resampled in register with the FC data. The final set of nuisance regressors also included the six parameters derived from the rigid-body head-motion correction, the global signal averaged over the (FreeSurfer-segmented) brain, and the global signal temporal derivative. Finally, the volumetric time series were nonlinearly warped to the Montreal Neurological Institute 152-space (3 mm)^3^ voxels using FNIRT (Jenkinson et al., 2012).

### Functional Connectivity

**Figure 1.**
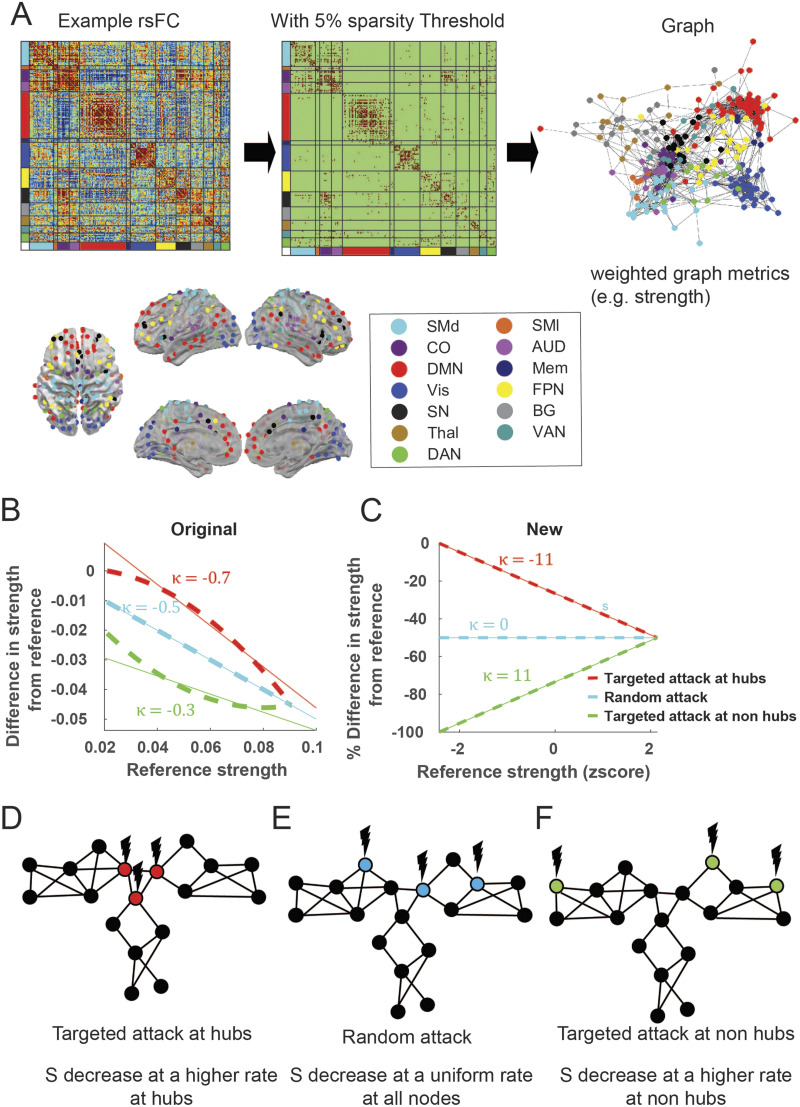
Graph theory method and hub disruption. (A) A resting-state FC (rsFC) is obtained from the Pearson correlation of the time series in each of the 246 cortical and subcortical predefined ROI pairs. The ROIs belong to 13 networks: SMd, somatomotor dorsal; SMl, somatomotor lateral; CO, cingulo-opercular; AUD, auditory; DMN, default mode network; Mem, memory network; Vis, visual network; FPN, frontoparietal network; SN, salience network; BG, basal ganglia; Thal, thalamus; VAN, ventral attention network; DAN, dorsal attention network. Following the convention in the previous literature, a sparse graph is generated by thresholding the rsFC matrix at an edge density threshold of 5% starting from the MST backbone to ensure the connectedness of the graph. However, to demonstrate that our results are not limited to the threshold choice, we also applied other thresholds. The graph generated has weighted edges that preserve the strength of individual connections. (B) Original method of hub disruption calculation. (C) A new method of hub disruption calculation. (D) Cartoon illustration of the targeted attack at the hubs. (E) Cartoon illustrating a random attack. (F) Cartoon illustration of the targeted attack at the non-hubs.

**Figure 2.**
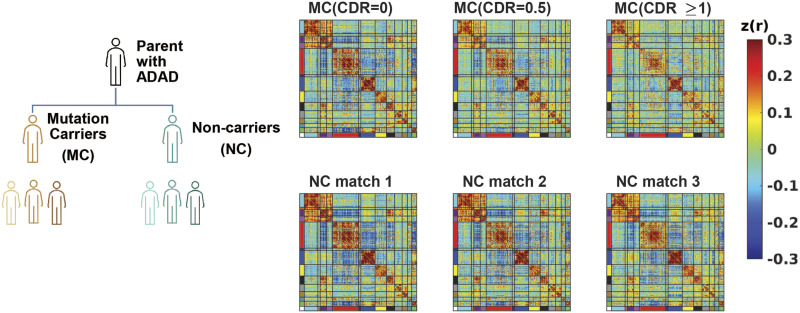
FC within the DIAN participant groups. Mean (lower triangle) and standard deviation (upper triangle) of the Fisher *Z*-transformed FC matrix of 246 ROIs for MC at three CDR stages (CDR = 0, CDR = 0.5, CDR ≥ 1) and corresponding age- and EYO-matched NC groups. The FC is sorted by the networks in Figure 1 with corresponding colors.

We selected 246 functional regions of interest (ROIs) separated into 13 networks throughout the cortical and subcortical areas as previously described (Wheelock et al., 2023). Functional ROIs are a combination of cortical ROIs (Power et al., 2011) and subcortical ROIs (Seitzman et al., 2020) (Figure 1A). Regions not reliably covered by the field of view such as the cerebellar ROIs were excluded. A list of ROI coordinates and anatomical assignments has been described in previous publications (Strain et al., 2022; Wheelock et al., 2023) and can be found in the Supporting Information Table S3. FC was estimated using zero-lag Pearson correlations calculated between 246 ROIs and Fisher *Z*-transformed. The resultant FC matrix can be represented as a graph with nodes as individual ROIs and edges with weights as the correlation values *z*(*r*). Group-averaged FC was then generated by averaging the *z*(*r*) values across individual FC matrices within each of the MC and age-matched NC groups (Figure 2). In addition, the gray matter volume variation across regions might affect the FC magnitude. Thus, we repeated our analysis after regressing out the gray matter volume for each ROI in each participant.

### Data Harmonization

We used Correcting Covariance Batch Effects (CovBat; https://github.com/WheelockLab/CovbatMatlabWrapper) (Chen et al., 2022) to remove site effects in mean, variance, and covariance on FC matrices, with age, mutation, EYO, education, CDR, sex, mutation gene type (PSEN1/PSEN2/APP), and Apolipoprotein E (APOE) alleles included as biological covariates to be protected during the removal of site effects. During CovBat, two participants (one MC and one NC) were removed from the analysis because they were only represented by a single site and harmonization could not be performed by the CovBat algorithm. The final sample size for analysis was MC = 121 and NC = 84. Similar qualitative results were obtained without the CovBat correction.

### Graph Theory Metrics

**Figure 3.**
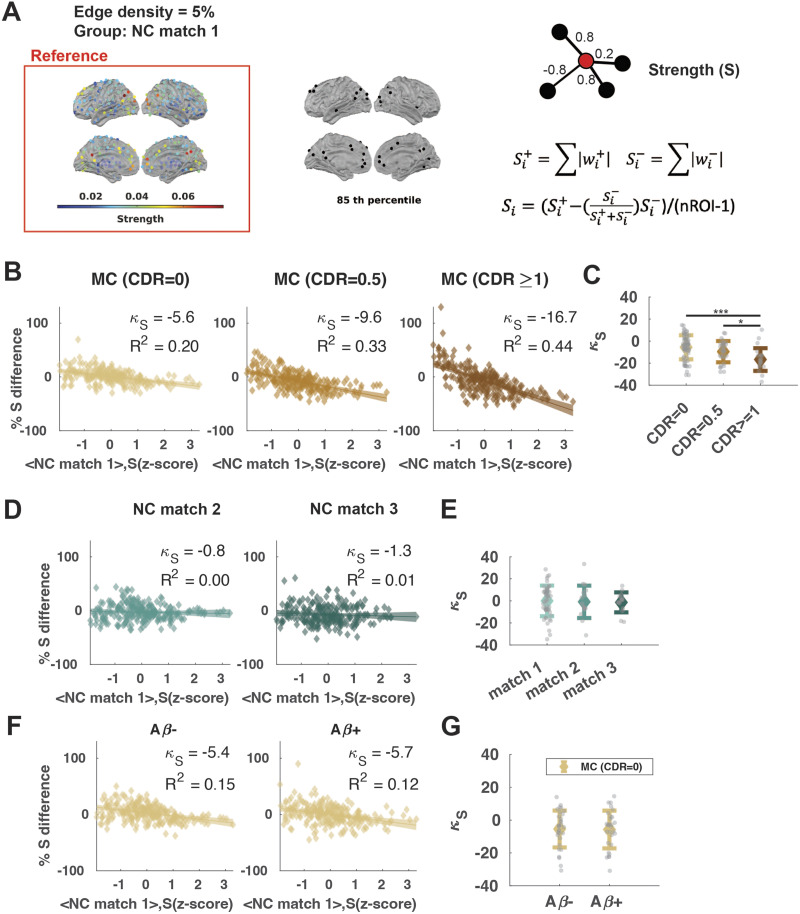
Hub disruption across CDR stages. (A) (Left) Distribution of average strength (*S*) across the NC match 1 group, (middle) nodes with *S* higher than the 85th percentile, and (right) cartoon illustrating that strength is calculated by summing the weights across the connected edges. (B) The percentage of *S* difference against the reference *S Z*-score in MC groups. (C) Individual hub disruption index (*κ_S_*) for MC groups. (D) The percentage of *S* difference against the reference *S Z*-score in NC groups. (E) Individual *κ_S_* for NC groups. (F) The percentage of *S* difference against the reference *S Z*-score in the subsets of A*β*− and A*β*+ participants in the MC (CDR = 0) group. (G) Individual *κ_S_* for A*β*− and A*β*+ participants in the MC (CDR = 0) group. Shaded areas show a 95% confidence interval. Error bars show mean and standard deviation. *p < 0.05, **p < 0.01, ***p < 0.001; FDR-corrected.

All graph theory metrics were calculated using the Brain Connectivity Toolbox (BCT; www.brain-connectivity-toolbox.net), a MATLAB toolbox for complex brain network analysis (Rubinov & Sporns, 2010). Since regions with a high total positive connectivity tend to have a high total negative connectivity (Supporting Information Figure S2), we asymmetrically weighted the positive and negative edges for all measures according to their relative magnitude at a given node, as described in previous literature (Rubinov & Sporns, 2011) (Figure 3A, Supporting Information Figure S3). This allows for nonzero strengths at a full FC matrix without a threshold. Strength is calculated as this asymmetrically weighted sum of signed edge weights around a node in a graph, divided by (number of ROIs −1) (Figure 1E). This effectively measures global connectivity at an ROI. We also calculated two additional measures of centrality concerning module affiliations (Guimerà & Nunes Amaral, 2005): the within-module strength *Z*-score (*Z*) and participation coefficient (Pc). *Z* measures how “well-connected” a node is to other nodes in the module. The Pc measures the diversity of intermodular connections of individual nodes and within-module strength *Z*-score.

Given that there is no gold standard method for thresholding the FC matrix to create a graph representation and calculate graph metrics (Drakesmith et al., 2015; Garrison et al., 2015), we chose an edge density threshold of 5% for downstream analyses, ensuring that the graph is sparse and free of negative correlations (Power et al., 2013). To demonstrate that our results are not dependent on the choice of threshold, we also showed results at a range of edge densities similar to previous research (Brier et al., 2014): 1%–5% at 1% intervals and 10%–90% at 10% intervals. This is achieved by finding the maximum spanning tree (MST) backbone first using the BCT toolbox function (backbone_wu.m) to ensure graph connectedness at the sparsest densities and continually adding edges with the largest correlation values until the desired edge density is reached (Hagmann et al., 2008).

Since the strength and Pc measures show a correlation with the scan duration of the retained frames, this was regressed out of these graph metrics to correct for the possible confound of individual differences in the total scan time remaining after frame censoring (Supporting Information Figure S4).

### Hub Disruption Index

To measure how the centrality of each region differs from a healthy reference, we chose NC match 1 to be the reference group. This choice was motivated by the fact that this group is the closest to what is usually considered as young, healthy adult controls (Dai et al., 2015; Yu et al., 2017), which has been used as a reference for calculating hub disruption in prior studies (Yu et al., 2017). The average nodal FC strength of the NC match 1 group was calculated, and the percentage difference from this reference average strength was calculated for each MC group (CDR = 0, CDR = 0.5, CDR ≥ 1; Figure 3B) and for the remaining two NC groups (NC match 2, NC match 3). While using a consistent reference group enables the comparison of the metric across groups (Achard et al., 2012), we also ran a supplementary analysis using the age-matched NC groups as a reference for each MC group and obtained qualitatively the same result.

The primary method by which selective hub disruption has been indexed in prior work was to calculate the slope of the linear regression model between the mean local network measures of a reference group, and the difference between that reference and the participant under study (Achard et al., 2012; Song et al., 2015; Termenon et al., 2016; Vatansever et al., 2020; Yu et al., 2017) (Figure 1A). A negative slope has previously been interpreted as the selective disruption at hubs. In practice, this phrasing is inaccurate and misleading. This is because even if strength (*S*) decreases at a uniform rate at all nodes (random attack) or at a higher rate at non-hubs (targeted attack at non-hubs), the original hub disruption index would still be negative (Figure 1B) since the absolute difference is highly dependent on the magnitudes of the reference. An intuitive analogy is to examine the effect of a natural disaster on the affluent versus impoverished areas. While an affluent area may suffer greater economic losses in absolute amounts, it is unclear whether that loss is attributable to incurring disproportionally more damage, or whether the loss reflects having started from a higher baseline. We, therefore, modified the “hub disruption index” to measure the *percentage* difference in connectivity strength versus reference connectivity strength such that only targeted attacks on hubs would result in a negative linear regression slope (Figure 1C). We calculated a normalized hub disruption index adapted from prior work (Achard et al., 2012) by fitting a linear regression slope (*κ*) and intercept (*b*) where the dependent variable was the difference in strength (*S*) for either the group average or an individual (*S*_test_) from the average *S* of the reference group (<*S*_*ref*_>), which was then normalized by dividing the average *S* of the reference group(<*S*_*ref*_>). The independent variable was the standardized *Z*-score of the reference *S* (*Z-score*(<*S*_*ref*_>)). In this way, we can unambiguously test the selective reduction in *S* at hubs and compare the hub disruption index quantitatively across disease stages. A more negative hub disruption index here indicates that the strengths in high-strength hubs are reduced by a larger proportion than in other low-strength regions, whereas a zero-hub disruption index indicates that the strength in high-strength regions and low-strength regions are changed to the same extent. The mathematical equation is shown below:

y=κx+b+error (1)

where *y* = $\frac{S_{\text{test}}-<S_{ref}>}{<S_{ref}>}\times 100$ and *x* = *Z-score*(<*S*_*ref*_>).

We used global connectivity strength as the primary measure of centrality due to its simplicity and strong correspondence to other measures in AD disease factors, for example, hubs with a high global FC have spatial correspondence with amyloid deposition (Buckner et al., 2009; Palmqvist et al., 2017; Villain et al., 2012), tau burdens (Cope et al., 2018), and metabolic factors (Liang et al., 2013; Vaishnavi et al., 2010). However, alternative definitions for hubs have been mentioned (Power et al., 2013; Sporns et al., 2007; van den Heuvel & Sporns, 2013). Specifically, some researchers argued that in functional networks, the Pc, which captures the diversity of connections to different modules, was the key metric for regional importance or centrality (Power et al., 2013). On the other hand, within-module strength *Z*-score is also an important measure of nodal centrality (Guimerà & Nunes Amaral, 2005). Therefore, we additionally examined hub disruption as defined by the Pc and within-module *Z*-score using a similar asymmetric weighting (Rubinov & Sporns, 2011) with the BCT toolbox functions (participation_coef_sign.m) and custom MATLAB scripts, respectively.

### Cognitive Composite Score

We used a cognitive composite score (CCS) (Aschenbrenner et al., 2020; Bateman et al., 2017; Wang et al., 2018), developed for use as an outcome measure in DIAN clinical trials, to measure the cognitive decline of each individual. CCS is a global summary of cognitive functions. Details of the calculation have been previously described (Wang et al., 2018). Briefly, a CCS is calculated by averaging each test’s normalized scores by equal weight for (a) the DIAN Word List test delayed recall, (b) the delayed recall score from the Wechsler Memory Scale—Revised Logical Memory IIA subtest, (c) the Mini-Mental State Exam, and (d) the Wechsler Adult Intelligence Scale—Revised Digit-Symbol Substitution test (Supporting Information Table S4). Normalization was carried out with respect to the mean and standard deviation reported in a population sample of 58 MCs with EYO ≤ −15 (Wang et al., 2018). For analyses using the CCSs, we excluded one MC with greater than 1-year gap between psychometric tests and MRI sessions, and additionally, nine MCs who did not complete all four tests.

### Positron Emission Tomography (PET) Measures of Cortical Amyloid Deposition

A*β* PET imaging with Pittsburgh Compound B (PiB) was performed using a bolus injection of [11C] PiB (McKay et al., 2023). PET data were acquired using either a 70-min scan beginning at the start of the injection or a 30-min scan starting 40 min after the injection. Data were converted to regional standardized uptake value ratios (SUVRs) relative to the cerebellar grey matter using the ROI generated in FreeSurfer (Fischl, 2012) with partial volume correction via a regional spread function. Amyloid positivity was defined as a PiB partial volume-corrected SUVR across the precuneus, prefrontal, gyrus rectus, and temporal FreeSurfer ROI > 1.42 (Brier et al., 2016; Su et al., 2013).

### Statistical Models for Biomarkers

Generalized additive mixed models were fit with the *gamm()* function from the R package (*mgcv*) to examine the relationship between different clinical markers and the EYO. A smooth function was applied to the EYO separated by mutation carrier status (MC or NC), with sex and education as fixed effect covariates, and a random intercept for family. When examining the relationship between the EYO and the hub disruption index, we additionally included the average FD of retained frames to control for the effect of motion. The time of divergence between the MC and the NC was determined as the EYO (to the nearest 0.1 years) where the predicted 83.4% simultaneous confidence interval started to have no overlap. The 83.4% confidence interval was considered more appropriate to assess the difference between two means as opposed to the difference between a mean and a point estimate; this gave a type I error rate of around *α* = 0.05 when the standard errors of the samples are similar (Knol et al., 2011; Payton et al., 2003). For this analysis, our sample consisted of the subset of 91 MCs and 80 NCs with both valid PiB and valid CCS measures.

For the relationship between CCS and *κ_S_*, we fit a linear mixed effects model with fixed effect covariates sex, education, average FD of retained frames, age, and a random intercept for the family with the *lmer()* function from R package *lme4*. For this analysis, our sample consisted of the 112 MCs with a valid CCS.

### Statistical Tests and Visualization

All standard statistical tests (e.g., *F* tests, *t* tests, analysis of variance) were performed with MATLAB R2020b or R (4.1.0). False discovery rate (Benjamini & Hochberg, 1995) was used for the correction of multiple comparisons at a significance level of 0.05.

Visualizations were generated using functions from the Network Level Analysis toolbox (Beta version) (https://github.com/mwheelock/Network-Level-Analysis), the BrainNet Viewer toolbox (Xia et al., 2013), and custom MATLAB and R scripts.

## RESULTS

### MC and NC Groups Do Not Differ in Demographic Features and Data Quality

As designed, each of the MC-matched and NC-matched CDR groups did not differ in age or EYO (Table 1). The matched groups also did not differ in average FD in retained frames or minutes of low-motion data. Moreover, no group differences were found between the MC groups and their NC matches except for minor differences in family mutation (*p* = 0.043) and education (*p* = 0.023) between the MC CDR = 1 and NC match 3. Not surprisingly, the MC CDR 0 group and NC match 1 did not differ on CCS, and CCS was significantly lower for the MC CDR = 0.5 and MC CDR ≥ 1 compared with NC match 2 and NC match 3, respectively.

### A Selected Subset of ROIs Shows Significant Differences in Strength From the Healthy Reference

We defined the average strength of ROIs in the young cognitively normal NC group (NC match 1, *N* = 52) as a reference of hub centrality (Figure 3A). Notably, we were able to identify the hubs described in the literature (Brier et al., 2014; Buckner et al., 2009; Cole et al., 2010), for example, precuneus/posterior cingulate, dorsolateral prefrontal cortex, supramarginal gyrus, and medial prefrontal cortex (Figure 3A; Supporting Information Figure S5). This was robust to the choice of edge density threshold and/or percentile cutoffs (Supporting Information Figure S6). Additionally, we compared the ROI strengths between all MC groups and the reference using a two-sample *t* test (FDR-adjusted *p* < 0.05) (Supporting Information Figure S7). Briefly, no ROI had a significant difference in strength between the MC (CDR = 0) and the reference. In the MC (CDR = 0.5), 19 ROIs covering the superior frontal gyrus, precuneus, middle temporal gyrus, middle occipital gyrus, middle frontal gyrus, inferior parietal lobule, inferior occipital gyrus, fusiform gyrus, and cuneus had significantly lower strength compared with the reference. In the MC (CDR ≥ 1), three ROIs (in the insula, thalamus, and parahippocampal gyrus) showed significantly higher strength compared with the reference, and 30 ROIs (in the angular gyrus, anterior cingulate, claustrum, cuneus, fusiform gyrus, inferior parietal lobule, inferior temporal gyrus, insula, medial frontal gyrus, middle occipital gyrus, middle temporal gyrus, parahippocampal gyrus, postcentral gyrus, posterior cingulate, precuneus, superior frontal gyrus, superior temporal gyrus, and thalamus) showed significantly lower strength compared with the reference. On the other hand, none of the ROIs in NC match 2 or NC match 3 groups showed significant differences in strength from NC match 1 (Supporting Information Figure S8).

### Hub Disruption Increases With the CDR Stage, Not Age

We measured the group-level hub disruption index by calculating the percentage difference from the reference for the mean strength in each of the MC groups (CDR = 0, CDR = 0.5, and CDR ≥ 1) (Figure 3B; Supporting Information Figure S9). The group-level hub disruption index for all three MC CDR groups was significantly different from 0 (Table 2). In addition, *κ_S_* became increasingly more negative across CDR stages. The hub disruption index (a.k.a. regression slope in Figure 3B) was significantly different between MC (CDR = 0.5) and MC (CDR = 0) (*F*(1, 488) = 12.0, *p* < 0.001, partial *η*^2^ = 0.024), between MC (CDR = 0.5) and MC (CDR ≥ 1) (*F*(1, 488) = 22.6, *p* < 0.001, partial *η*^2^ = 0.044), and between MC (CDR = 0) and MC (CDR ≥ 1) (*F*(1, 488) = 61.6, *p* < 0.001, partial *η*^2^ = 0.112). Our results were qualitatively replicated at a wide range of threshold choices (Supporting Information Figure S10). On average, nodes in the cingulo-opercular network showed the highest reference strength and largest percentage of strength difference from reference across multiple thresholds (Supporting Information Figure S11). In addition to the group-level hub disruption index, we calculated the hub disruption index for each participant in the MC and NC groups. All MC groups had a hub disruption index that differed from 0 (FDR-adjusted *p* < 0.001), while no NC group had a hub disruption index that differed from 0 (FDR-adjusted *p* > 0.05) (Supporting Information Figure S12, Supporting Information Table S5). Specifically, for the MC, a one-way ANOVA demonstrated that the hub disruption index differed across the CDR groups (*F*(2, 118) = 8.8, *p* < 0.001, *η*^2^ = 0.130). Post hoc two-sample *t* tests with FDR correction revealed significant group differences (*t*(87) = 4.03, *p* < 0.001, Cohen’s *d* = 1.02) between CDR = 0 (*M* = −5.6, *SD* = 11.1) and CDR ≥ 1 participants (*M* = −16.7*, SD* = 10.3) and between CDR = 0.5 (*M* = −9.6, *SD* = 9.6) and CDR ≥ 1 participants (*t*(50) = 2.52, *p* = 0.03, Cohen’s *d* = 0.72) (Figure 3C).

**Table 2.** Group-level hub disruption (using the metrics in NC match 1 as a baseline) across CDR stages in the MC and across age in the NC (FDR-adjusted)

Strength (*S*)					
	Group	*κ_S_*	*F*(1, 244)	*p*	*R* ^2^
MC	CDR = 0	−5.6	59.1	<0.001	0.20
	CDR = 0.5	−9.6	118.1	<0.001	0.33
	CDR ≥ 1	−16.7	192.2	<0.001	0.44
NC	Match 2	−0.8	0.78	0.38	0.003
	Match 3	−1.3	1.46	0.28	0.006
Participation coefficient (Pc)					
	Group	*κ_S_*	*F*(1, 244)	*p*	*R* ^2^
MC	CDR = 0	3.3	17.8	<0.001	0.07
	CDR = 0.5	1.7	2.5	0.19	0.01
	CDR ≥ 1	−1.6	1.0	0.34	0.44
NC	Match 2	2.1	4.7	0.08	0.02
	Match 3	−1.0	0.9	0.34	0.003
Within-module strength *Z*-score (*Z*)					
	Group	*κ_S_*	*F*(1, 244)	*p*	*R* ^2^
MC	CDR = 0	−5.0	12.6	<0.001	0.05
	CDR = 0.5	−7.2	15.5	<0.001	0.06
	CDR ≥ 1	−12.7	22.1	<0.001	0.08
NC	Match 2	−2.1	1.6	0.21	0.01
	Match 3	−3.3	2.9	0.11	0.01

Next, we investigated whether this observation can be explained by increasing age. We calculated the hub disruption index for the age-matched NC groups 2 and 3 with the same procedure (Figure 3D). The group-level hub disruption index for NC groups did not significantly differ from 0 (Table 2). At the individual level, there were no differences among NC groups (one-way ANOVA, *F*(2, 81) = 0.07, *p* = 0.930) (Figure 3E) nor was a significant relationship between *κ_S_* and age in NC (linear regression, *β* = 0.01, *R*^2^ < 0.001, *F*(2, 82) = 0.0072, *p* = 0.933).

Changes in A*β* accumulation in PET imaging often precede dementia symptoms in AD (Bateman et al., 2012; Jack et al., 2010; Sperling et al., 2011). A subset of the MC (CDR = 0) group can be classified as A*β* positive (A*β*+) (*N* = 29) according to their amyloid PET results (see the Materials and Methods section). With one-sample *t* tests with FDR correction, we found that both groups have a hub disruption index significantly lower than 0 (A*β*+: *M* = −5.7, *SD* = 11.5, Cohen’s *d* = −0.50, *t*(28) = −2.6, *p* = 0.012; A*β*−: *M* = −5.4, *SD* = 11.2, Cohen’s *d* = −0.48, *t*(30) = −2.7, *p* = 0.012). However, there was no significant difference in the hub disruption index between the A*β*+ and A*β*− groups (two-sample *t* test, *t*(58) = −0.11, *p* = 0.920, Cohen’s *d* = −0.03) (Figure 3F–G).

Given that some of the regions with the biggest difference in cortical atrophy between symptomatic MC and NC participants (Gordon et al., 2018) also have high centrality, the hub disruption in FC that we observed might be partially explained by cortical atrophy. We have also repeated our analysis after regressing out the gray matter volume for each ROI in each participant and have obtained the same results (Supporting Information Figure S13).

### Hub Disruption Is Best Explained by Differences in Global Connectivity

**Figure 4.**
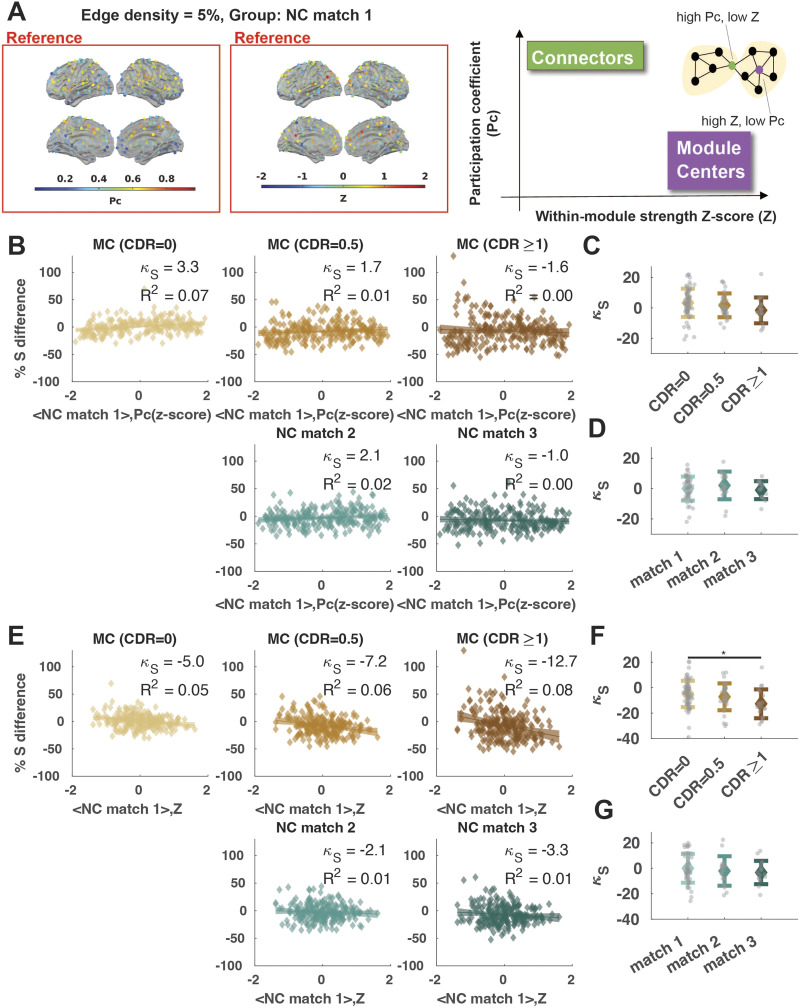
Hub disruption across CDR stages at module centers versus connectors. (A) (Left) Distribution of the average participation coefficient (Pc) across the NC match 1 group, (middle) distribution of within-strength *Z*-score (*Z*) across the NC match 1 group, and (right) cartoon illustrating the representation of module centers and connectors on a graph. Module centers are nodes with a high *Z*-score, and connectors are nodes with a high Pc. (B) The percentage of *S* difference against the reference Pc *Z*-score for hub disruption calculation. (C) Individual hub disruption index (*κ_S_*) for the MC with respect to the group average Pc *Z*-score at NC match 1. (D) Individual hub disruption index (*κ_S_*) for the NC with respect to the group average Pc *Z*-score at NC match 1. (E) The percentage of *S* difference against the reference *Z* for hub disruption calculation. (F) Individual hub disruption index (*κ_S_*) for the MC with respect to the group average *Z* at NC match 1. (G) Individual hub disruption index (*κ_S_*) for the NC with respect to the group average *Z* at NC match 1. Lines show the linear fit and shaded areas indicate the 95% confidence interval. *p < 0.05, **p < 0.01, ***p < 0.001; FDR-corrected.

To understand the key drivers of hub vulnerability in ADAD, we calculated the hub disruption index using two alternative measures based on their network membership instead of global connectivity strength: (a) the within-module connectivity rank (within-module strength *Z*-score, *Z*) and (b) the connectivity diversity (participation coefficient, Pc) (Figure 4A). Overall, both Pc and within module *Z*-score effects were less sensitive to ADAD progression than using the global connectivity strength as the reference. Thus, we focused our subsequent analyses on the hub disruption index in relation to the global connectivity strength. Detailed statistics can be found in Table 2, Supporting Information Appendix A, and Supporting Information Figures S14–S19.

### Hub Disruption Predates Cognitive Changes But Follows Amyloid PET Changes

**Figure 5.**
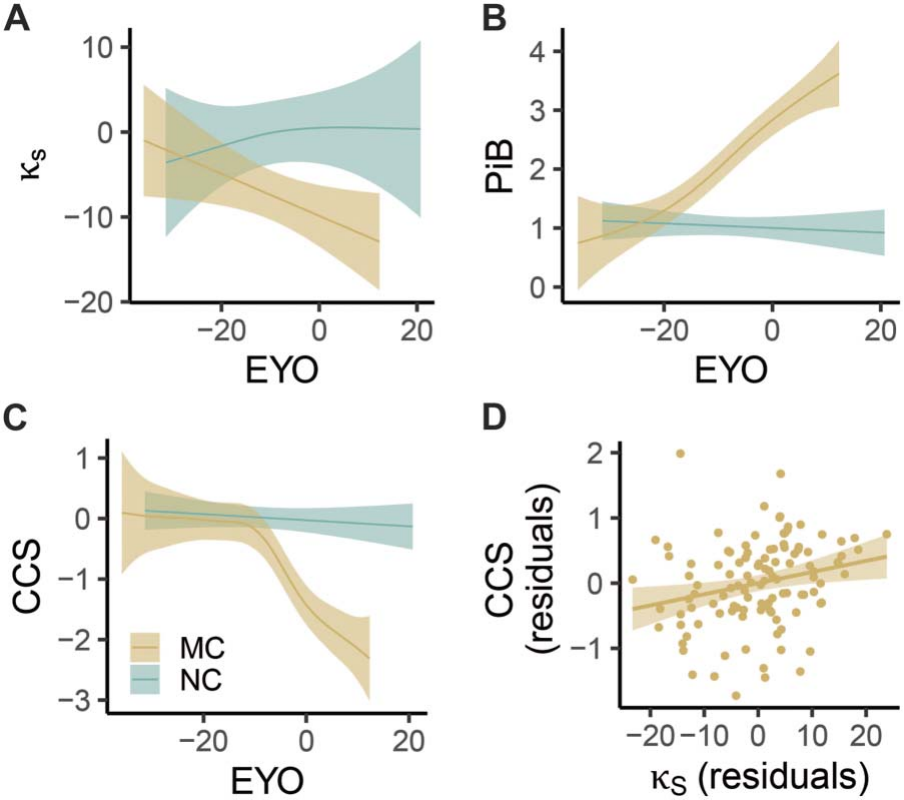
Change in biomarkers across the estimated EYO for the MC and the NC. (A) The partial effect of EYO on the hub disruption index in strength (*κ_S_*). (B) The partial effect of EYO on total cortical amyloid deposition measured with PiB. (C) The partial effect of EYO on CCS. The line and shaded areas show the predicted response values and the confidence intervals for the fitted responses from a generalized additive model at a 95% Bayesian credible interval. For privacy reasons, individual data points including EYO were not displayed but were used in model fitting. (D) The CCS against *κ_S_* after regressing out potential confounding variables from both.

Generalized additive mixed models were fit to examine the relationship between hub disruption or other biomarkers and the EYO, as well as to obtain the point of divergence between MC and NC. For the hub disruption index (*κ_S_*), this was calculated to be EYO = −11.7 years (Figure 5A). In comparison, the total cortical amyloid deposition measured as PiB SUVR diverged at EYO = −16.9 (Figure 5B), and the CCS measure diverged at EYO = −7.3 years (Figure 5C). Thus, we found that the divergence of the hub disruption index preceded the divergence of cognitive performance measures and followed the earlier stage of amyloid deposition. However, the differences in the divergence points between *κ_S_* and PiB (*p* = 0.218) or between *κ_S_* and CCS (*p* = 0.154) were not significant based on 1,000 bootstrap samples with a valid divergent point. As expected, given their correspondence with disease progression, there was a negative correlation (*r* = −0.36, *p* < 0.001) between PiB and *κ_S_*, but this relationship was not significant within the CDR = 0 participants (Supporting Information Figure S20).

In addition, to examine the performance of *κ_S_* in distinguishing MC individuals from mutation NC individuals at different ranges of EYO (Supporting Information Table S9) and age (Supporting Information Table S10), we calculated the area under the curve in 5-year bins and observed that AUC ranges from 0.53 to 0.83 across EYO and 0.60 to 0.87 across age bins.

### Greater Hub Disruption Is Correlated With Worse General Cognition

Lastly, we found that there existed a positive correlation between *κ_S_* and CCS (*r* = 0.3, *t*(110) = 3.27, *p* = 0.001). We further examined whether an individual’s hub disruption could explain the unique variance in the CCS of individual MCs after controlling for potentially confounding covariates (age, sex, years of education, motion in scan measured by the average FD of the retained frames as fixed effect, and a random intercept for family). The hub disruption index was positively associated with CCSs at the edge threshold of 5% (*β*_*κ*_S_ = 0.02 ± 0.01, *t*(105) = 2.52, *p* = 0.013) and across different edge thresholds (Table 3, Figure 5D), suggesting that greater hub disruption (a.k.a. more negative hub disruption index) correlated with worse general cognition.

**Table 3.** Regression of the hub disruption index on CCS

Edge threshold	*β_κ_S_*	*β_Education*	*β_Age*	*β_Sex (Male)*	*β_FD*
1%	0.02	0.11***	−0.06***	−0.16	−2.72
2%	0.01*	0.11***	−0.06***	−0.15	−2.48
3%	0.01*	0.11***	−0.06***	−0.16	−2.36
4%	0.02*	0.11***	−0.06***	−0.16	−2.38
5%	0.02*	0.11***	−0.06***	−0.16	−2.32
10%	0.03**	0.11***	−0.06***	−0.16	−2.37
20%	0.05**	0.11***	−0.06***	−0.16	−2.57
30%	0.07**	0.11***	−0.06***	−0.17	−2.68
40%	0.09**	0.10***	−0.05***	−0.18	−2.77

Response: CSS, cognitive composite scores; *β*, coefficient of regression. *p < 0.05, **p < 0.01, ***p < 0.001. Random effect: Family.

## DISCUSSION

We investigated the relationship between FC differences across ROIs and reference centrality measures. Consistent with a targeted attack on hubs model, the proportion of reduction in FC at individual regions in ADAD was positively related to the global connectivity strength of that region in the unaffected family members of ADAD participants. This preferential disruption of hub connectivity increased with CDR stage but not age was best explained by global connectivity, less so by the within-module connectivity rank, and not by the diversity of connectivity across resting-state networks. This preferential disruption of hub connectivity was seen at all stages of disease progression in the ADAD MC and started to significantly differentiate MC and NC at about 12 years before the expected clinical symptoms, predating cognitive changes but following amyloid PET changes, indicating the early and progressive nature of hub vulnerability in AD. Additionally, greater (more negative) hub disruption was associated with worse general cognition after controlling for relevant covariates. These findings provided insights into the complex dynamics of brain network dysfunction in AD and the critical role of hubs in this process.

### Progressive Hub Disruption Is Consistent With Popular Network Failure Models of AD

Prior studies endorsed a cascading network failure starting from the posterior DMN and progressing to the anterior and ventral DMN (Jones et al., 2016). Our results complemented this observation by providing a possible underlying factor driving this cascading process: The vulnerability of regions to the reduction in FC is dependent on their centrality in the whole brain network. Nodes that have the highest strength in the FC network (e.g., posterior DMN) are among the first to show decreased FC, consistent with existing literature (Jones et al., 2016). Over time, changes in processing burden shift from one hub to other hubs, further enhancing the aberrant A*β* precursor protein processing and amyloidosis in the other hubs (Jones et al., 2016), consistent with our progressive increase in hub disruption with disease. Prior work simulating activity-dependent degeneration observed decreased structural connectivity throughout disease progression while FC first increased and then decreased (de Haan et al., 2012). Similarly, the cascading network failure model of AD also characterized an initial increase in FC (Jones et al., 2016). We did not observe significantly higher *S* in MCI compared with healthy controls, and three non-hub ROIs have significantly higher *S* in AD compared with healthy NC, which might be due to the lack of power of the current study or limitation of the theory. Future research could investigate hub vulnerability in structural networks using diffusion MRI for proper interpretation of activity-dependent degeneration.

### Lower FC in ADAD Carriers Are Especially Dominant at Hubs With High Global Connectivity

Despite the wide use of “functional hubs” in the literature, what defines a functional hub has not reached a consensus (Power et al., 2013; Sporns et al., 2007; van den Heuvel & Sporns, 2013). Hubs can be described in terms of their network membership (e.g., DMN), where connectors are important for communication between networks and module centers are important for communication within networks. The nodes with a high abundance of intermodule connections (connectors) form a structural rich club (de Reus & van den Heuvel, 2013), which are also known to be affected in AD (Cao et al., 2020), although other studies suggested that the highly rich club core was preserved and the disruptions started in the periphery (Daianu et al., 2015). Previous literature on brain lesion patients suggested that the integrity of brain network organization was severely compromised when damage was in connectors but not in module centers (Gratton et al., 2012). Other studies also reported differential outcomes in network structure when damage was localized to module centers or connectors (Honey & Sporns, 2008). One recent study has also suggested that the A*β* accumulation rate was faster at connectors (Liu et al., 2023) Therefore, we examined whether the spatial pattern of FC difference from the reference group was explained by global connectivity, connectivity diversity across networks, or local connectivity within networks. Our results here suggested that the spatial distribution of FC difference across clinical stages was best explained by differences in global connectivity across regions, rather than by their roles to communicate between or within networks, consistent with the hypothesis of processing load shift to high connectivity hubs (Jones et al., 2017). This is in line with the hypothesis that high metabolic demands, associated with high global connectivity, may trigger downstream cellular and molecular events that result in neurodegeneration (Jones et al., 2016), conveying preferential/selective vulnerability. For example, calcium instability caused by A*β* peptides might render human cortical neurons vulnerable to excitotoxicity (Mattson et al., 1992), and this could result in further neurodegeneration in AD (Bezprozvanny & Mattson, 2008).

### Hub Disruption Predates Cognitive Changes But Follows Amyloid PET Changes

The effectiveness of a biomarker can be evaluated based on its ability to detect early indications of pathology before disease onset. Investigating the initial stages of decline in healthy brains compared with those with AD offers substantial potential for early identification before AD symptoms manifest. Because of the highly consistent familial disease onset for ADAD, we were able to compare this biomarker across EYO and other disease-related changes including CCSs and cortical amyloid deposition. We found that the hub disruption index first demonstrated a divergence between groups ~12 years before EYO—earlier than the divergence in the global FC signature (~4 years)—between converters and non-converters in sporadic AD (Wisch et al., 2020). The hub disruption index also diverges between MCs and NCs after the general cognitive score (~7 years), but after hypometabolism (~10 years), and increased concentrations of cerebral spinal fluid tau protein (~15 years) (Bateman et al., 2012), and the amyloid PET changes (~15 years). The divergence point calculated by bootstrap samples between the hub disruption index and other clinical markers were not significantly different, which might be due to the small sample size used in the analysis or the high noise/low reliability of the FC-based hub disruption index metric. This is consistent with the previously hypothesized disease progression where the FC disruption follows from A*β* deposition and potentially excessive chronic activity (de Haan et al., 2012; Hampel et al., 2021) and eventually contributes to cognitive impairment (Supporting Information Figure S21). However, we did not find significant differences between the hub disruption index in A*β*+ and A*β*− participants in the MC (CDR = 0) group, despite A*β*+ participants having a slightly more negative hub disruption index. However, this lack of a difference between A*β*− and A*β*+ individuals should be viewed cautiously given the modest sample sizes. We do note the limitation that our EYO calculation is based on mutation and parental symptom onset and may not precisely reflect the true EYO, but this would have an equal effect on all biomarkers. Therefore, the best practice is to interpret the EYO in relative terms for different biomarkers instead of taking it at purely its face value.

### Comparison With Other Network Topology Studies in AD

Other studies of network topology in AD have examined global graph theory measures such as small-worldness, global clustering coefficient, and characteristic path length (Brier et al., 2014; delEtoile & Adeli, 2017). However, those measures are generally sensitive to network sparsity (van Wijk et al., 2010) and require a careful choice of null models (Váša & Mišić, 2022), although recent studies have attempted to mitigate the problems using the minimum spanning tree measurements of graphs (Blomsma et al., 2022). Further, it is hard to interpret the biological relevance of those global measures. In contrast, hubs with a high global FC have been linked to amyloid deposition (Buckner et al., 2009; Palmqvist et al., 2017; Villain et al., 2012), tau burden (Cope et al., 2018), and metabolic factors (Liang et al., 2013; Vaishnavi et al., 2010). They also overlap with the regions that demonstrated high heritability (Fornito et al., 2011). Therefore, our research on hub vulnerability is driven by the literature with an attempt to link abstract network topology measures to molecular and cellular pathologies.

### Implications for AD Research, Prevention, and Treatment

We found that hub disruption, or increased vulnerability to reduced FC at highly central hub regions, was prevalent across the course of ADAD, with increasing severity as the disease progresses. Our results here have important implications for future AD research and therapeutics development: We provided a testable hypothesis where targeted pharmacological manipulation, noninvasive stimulation (Koch et al., 2022), or behavioral training to alter neuronal excitability (de Haan et al., 2017) especially at hub regions could potentially alter the progression of AD. Existing research has demonstrated in an awake rodent model that acute inactivation of a hub region (dorsal anterior cingulate cortex) has profound effects on the whole network (Tu et al., 2021). Future studies in animal models of AD could further validate this with optogenetic and chemogenetic manipulations. Furthermore, previous literature has suggested that “restoration of the topology of resting-state FC may aid in cognitive repair and recovery” (Alstott et al., 2009; Rubinov et al., 2009), and those can be further tested in future studies.

On the other hand, we found that hub disruption was positively related to the CCSs after considering the effect of age, sex, years of education, and average motion of the retained frame. The separation of hub disruption between the MC and NC starts shortly after the increased levels of cortical amyloid deposition and at around the same time as preclinical measures of cognitive decline. This indicates that our new measure of resting-state FC change has the potential to act as a noninvasive, low-cost, and accessible biomarker especially given compared with cerebrospinal fluid and PET for prevention studies and clinical trials to aid in the development of new treatments and monitor their effectiveness. Other biomarkers focusing on DMN failure have been proposed (Wiepert et al., 2017), but our measure is conceptually straightforward, easy to calculate, and biologically intuitive. In addition, previous measures have focused on distinguishing AD patients from controls, whereas the current study mapped a progressive relationship between FC and centrality across the clinical dementia stages.

### Limitations and Future Directions

One limitation of our study is that the in-scanner head motion tends to increase with the severity of dementia, and hence, the samples included in our analysis are biased toward participants with less severe symptoms. However, since we are most interested in the early changes in AD disease, this limitation has little impact on our conclusions.

In addition, while we concluded that increasing hub disruption is related to disease progression and not aging, participants involved in this study were relatively young (18–69 years old). It is still possible that a similar increase in hub vulnerability might be observed at a much older age, as seen in other age-related changes in FC (Jones et al., 2011; Wig, 2017). Notably, another study using cognitively normal adults from the OASIS-3 (Open Access Series of Imaging Studies; 42–95 years old) appeared to show results opposite to the current study (Liu et al., 2023), whereby functional hubs were particularly vulnerable to the higher annual accumulation of A*β* but have a slower FC decrease than non-hub regions. However, there are also several important methodological differences between that study and ours: (a) They employed the GLASSO Graphical LASSO (Mazumder & Hastie, 2012); algorithm to estimate the FC with only direct connections, whereas we used the simple Pearson’s correlation, and (b) they define hubs as regions with high Pc and we found that at a certain edge density threshold, the strength and the Pc of a node could be negatively correlated.

Additionally, even though previous work has found comparable FC changes in ADAD to sporadic AD (Smith et al., 2021; Strain et al., 2022; Wheelock et al., 2023), our findings have yet to be confirmed in sporadic AD. Further validations on longitudinal changes and subjects with more imaging data are needed to assess whether hub disruption could be a reliable biomarker of individual disease progression in AD. For example, one could examine whether the hub disruption index predicts the conversion of MCI to AD using measures such as accuracy or area under the curve of a ROC. In addition, while amyloid deposition is localized to high connectivity hubs, an elevated tau-PET signal appear simultaneously across resting-state networks (Jones et al., 2017). Previous modeling evidence suggested that tau might spread from focal epicenters across functional connections (Franzmeier et al., 2020) and that stronger tau deposition in globally connected hubs was associated with earlier AD symptom manifestation (Frontzkowski et al., 2022). Future studies could examine the interplay between amyloid, tau, and hub vulnerability in more detail. Furthermore, future investigations in large brain-wide single-cell transcriptome data (e.g., Allen Human Brain Atlas) may be useful in linking hub vulnerability to the underlying biological mechanisms (Diez & Sepulcre, 2021; Wagstyl et al., 2024).

While the entorhinal cortex and hippocampus are key areas that are implicated in AD, especially in the context of tau spreading (Braak & Braak, 1991), they appear to be less critical for tau spreading in ADAD (Gordon et al., 2018). We recognize the failure to capture those regions as a limitation of our study.

## ACKNOWLEDGMENTS

Data collection and sharing for this project were supported by the Dominantly Inherited Alzheimer Network (DIAN; U19AG032438) funded by the National Institute on Aging (NIA), the Alzheimer’s Association (SG-20-690363-DIAN), the German Center for Neurodegenerative Diseases (DZNE), Raul Carrea Institute for Neurological Research (FLENI), partial support by the Research and Development Grants for Dementia from Japan Agency for Medical Research and Development, AMED, and the Korea Health Technology R&D Project through the Korea Health Industry Development Institute (KHIDI), Spanish Institute of Health Carlos III (ISCIII), Canadian Institutes of Health Research (CIHR), Canadian Consortium of Neurodegeneration and Aging, Brain Canada Foundation, and Fonds de Recherche du Québec–Santé. This manuscript has been reviewed by DIAN Study investigators for scientific content and consistency of data interpretation with previous DIAN Study publications. We acknowledge the altruism of the participants and their families and the contributions of the DIAN research and support staff at each of the participating sites for their contributions to this study. During the preparation of this work, the author(s) used ChatGPT to increase the clarity and conciseness of the language. After using this tool/service, the author reviewed and edited the content as needed and took full responsibility for the content of the publication. We would like to additionally thank Aaron Tanenbaum for processing the MRI data; Dr. Julie Wisch, Dr. Tyler Blazey, and Dr. Matt Welhaf for their suggestions and assistance in estimating the divergence of metrics between MCs and NCs; Dr. Nilanjan Chakraborty, Sayan Das, Dr. Aishwarya Rajesh, and Jiaqi Li for their discussion on the mathematical rigor; as well as Dr. Timothy O. Laumann for discussions on the manuscript revision.

## SUPPORTING INFORMATION

Supporting information for this article is available at https://doi.org/10.1162/netn_a_00395.

## AUTHOR CONTRIBUTIONS

Jiaxin Cindy Tu: Conceptualization; Formal analysis; Funding acquisition; Methodology; Visualization; Writing – original draft; Writing – review & editing. Peter R. Millar: Methodology; Writing – review & editing. Jeremy F. Strain: Writing – review & editing. Andrew Eck: Software; Validation. Babatunde Adeyemo: Writing – review & editing. Abraham Z. Snyder: Formal analysis; Writing – review & editing. Alisha Daniels: Funding acquisition; Investigation; Writing – review & editing. Celeste Karch: Conceptualization; Funding acquisition; Investigation; Resources; Writing – review & editing. Edward D. Huey: Funding acquisition; Investigation; Resources; Writing – review & editing. Eric McDade: Funding acquisition; Investigation; Resources; Writing – review & editing. Gregory S. Day: Funding acquisition; Investigation; Resources; Writing – review & editing. Igor Yakushev: Writing – review & editing. Jason Hassenstab: Funding acquisition; Investigation; Methodology; Writing – review & editing. John Morris: Funding acquisition; Investigation; Resources; Writing – review & editing. Jorge J. Llibre-Guerra: Funding acquisition; Investigation; Resources; Writing – review & editing. Laura Ibanez: Funding acquisition; Investigation; Resources; Writing – review & editing. Mathias Jucker: Funding acquisition; Investigation; Resources; Writing – review & editing. Patricio Chrem Mendez: Funding acquisition; Investigation; Resources; Writing – review & editing. Richard J. Perrin: Funding acquisition; Investigation; Resources; Writing – review & editing. Tammie Benzinger: Funding acquisition; Investigation; Resources; Writing – review & editing. Clifford R. Jack Jr.: Funding acquisition; Investigation; Resources; Writing – review & editing. Richard Betzel: Writing – review & editing. Beau M. Ances: Funding acquisition; Investigation; Resources; Writing – review & editing. Adam T. Eggebrecht: Conceptualization; Supervision; Writing – review & editing. Brian A. Gordon: Funding acquisition; Investigation; Resources; Writing – review & editing. Muriah D. Wheelock: Conceptualization; Funding acquisition; Supervision; Writing – review & editing.

## FUNDING INFORMATION

Muriah D. Wheelock, NIH, Award ID: K99 EB029343. Jiaxin Cindy Tu, McDonnell Center for System Neuroscience, Washington University in St. Louis, Award ID: CCSN Fellowship. Muriah D. Wheelock, NIH, Award ID: R00EB029343. Brian A. Gordon, NIH, Award ID: K01 AG053474. Muriah D. Wheelock, NIH, Award ID: P30 AG066444.

## DATA AND CODE AVAILABILITY

Data that support the findings of this study are available from the DIAN consortium upon request at https://dian.wustl.edu/our-research/observational-study/dian-observational-study-investigator-resources/. The code is available on GitHub: https://github.com/WheelockLab/Tu-2024-DIAN-HubDisruption-FC.

## Supplementary Material



## TECHNICAL TERMS

Functional connectivity: Measured as the statistical dependence (here, Pearson’s correlation) between the time series of two network nodes.
Hub: A node occupying a central position in the overall organization of a network.
Centrality: The importance of a node in a network, the most common one being degree/strength.
Degree/strength: The sum of numbers/weights of edges (divided by *n* − 1 in the current study following Rubinov & Sporns, 2011).
Module: A group of nodes that maintain a large number of mutual connections and a small number of connections to nodes outside their module.
Participation coefficient: A graph theoretical measure that represents the diversity of connections across all modules in a network.
Maximum spanning tree: A subgraph with (*n* − 1) edges that connects all nodes of the network such that the sum of its weights is maximal, where n is the number of nodes.
Hub disruption index: A global index that represents the relationship between the new centrality of nodes and the reference centrality of nodes in a graph.
Connector: Nodes with a high Pc with even connectivity spanning a diverse profile of modules in a network.
Module center: Nodes with a high degree/strength compared with other nodes in the same module.

## References

[bib1] Achard, S., Delon-Martin, C., Vértes, P. E., Renard, F., Schenck, M., Schneider, F., … Bullmore, E. T. (2012). Hubs of brain functional networks are radically reorganized in comatose patients. Proceedings of the National Academy of Sciences, 109(50), 20608–20613. 10.1073/pnas.1208933109, 23185007 PMC3528500

[bib2] Achard, S., Salvador, R., Whitcher, B., Suckling, J., & Bullmore, E. (2006). A resilient, low-frequency, small-world human brain functional network with highly connected association cortical hubs. Journal of Neuroscience, 26(1), 63–72. 10.1523/JNEUROSCI.3874-05.2006, 16399673 PMC6674299

[bib3] Alstott, J., Breakspear, M., Hagmann, P., Cammoun, L., & Sporns, O. (2009). Modeling the impact of lesions in the human brain. PLOS Computational Biology, 5(6), e1000408. 10.1371/journal.pcbi.1000408, 19521503 PMC2688028

[bib4] Aschenbrenner, A. J., James, B. D., McDade, E., Wang, G., Lim, Y. Y., Benzinger, T. L. S., … Dominantly Inherited Alzheimer Network. (2020). Awareness of genetic risk in the Dominantly Inherited Alzheimer Network (DIAN). Alzheimer’s & Dementia, 16(1), 219–228. 10.1002/alz.12010, 31914221 PMC7206736

[bib5] Bateman, R. J., Benzinger, T. L., Berry, S., Clifford, D. B., Duggan, C., Fagan, A. M., … DIAN-TU Pharma Consortium for the Dominantly Inherited Alzheimer Network. (2017). The DIAN-TU Next Generation Alzheimer’s prevention trial: Adaptive design and disease progression model. Alzheimer’s & Dementia, 13(1), 8–19. 10.1016/j.jalz.2016.07.005, 27583651 PMC5218895

[bib6] Bateman, R. J., Xiong, C., Benzinger, T. L. S., Fagan, A. M., Goate, A., Fox, N. C., … Dominantly Inherited Alzheimer Network. (2012). Clinical and biomarker changes in dominantly inherited Alzheimer’s disease. New England Journal of Medicine, 367(9), 795–804. 10.1056/NEJMoa1202753, 22784036 PMC3474597

[bib7] Behzadi, Y., Restom, K., Liau, J., & Liu, T. T. (2007). A component based noise correction method (CompCor) for BOLD and perfusion based fMRI. NeuroImage, 37(1), 90–101. 10.1016/j.neuroimage.2007.04.042, 17560126 PMC2214855

[bib8] Benjamini, Y., & Hochberg, Y. (1995). Controlling the false discovery rate: A practical and powerful approach to multiple testing. Journal of the Royal Statistical Society: Series B (Methodological), 57(1), 289–300. 10.1111/j.2517-6161.1995.tb02031.x

[bib9] Bero, A. W., Yan, P., Roh, J. H., Cirrito, J. R., Stewart, F. R., Raichle, M. E., … Holtzman, D. M. (2011). Neuronal activity regulates the regional vulnerability to amyloid-β deposition. Nature Neuroscience, 14(6), 750–756. 10.1038/nn.2801, 21532579 PMC3102784

[bib10] Bezprozvanny, I., & Mattson, M. P. (2008). Neuronal calcium mishandling and the pathogenesis of Alzheimer’s disease. Trends in Neurosciences, 31(9), 454–463. 10.1016/j.tins.2008.06.005, 18675468 PMC2566585

[bib11] Biswal, B., Yetkin, F. Z., Haughton, V. M., & Hyde, J. S. (1995). Functional connectivity in the motor cortex of resting human brain using echo-planar MRI. Magnetic Resonance in Medicine, 34(4), 537–541. 10.1002/mrm.1910340409, 8524021

[bib12] Blomsma, N., de Rooy, B., Gerritse, F., van der Spek, R., Tewarie, P., Hillebrand, A., … van Dellen, E. (2022). Minimum spanning tree analysis of brain networks: A systematic review of network size effects, sensitivity for neuropsychiatric pathology, and disorder specificity. Network Neuroscience, 6(2), 301–319. 10.1162/netn_a_00245, 35733422 PMC9207994

[bib150] Braak, H., & Braak, E. (1991). Neuropathological stageing of Alzheimer-related changes. Acta Neuropathologica, 82(4), 239–259. 10.1007/BF00308809, 1759558

[bib13] Brier, M. R., MCarthy, J. E., Benzinger, T. L. S., Stern, A., Su, Y., Friedrichsen, K. A., … Vlassenko, A. G. (2016). Local and distributed PiB accumulation associated with development of preclinical Alzheimer’s disease. Neurobiology of Aging, 38, 104–111. 10.1016/j.neurobiolaging.2015.10.025, 26827648 PMC5279747

[bib14] Brier, M. R., Thomas, J. B., Fagan, A. M., Hassenstab, J., Holtzman, D. M., Benzinger, T. L., … Ances, B. M. (2014). Functional connectivity and graph theory in preclinical Alzheimer’s disease. Neurobiology of Aging, 35(4), 757–768. 10.1016/j.neurobiolaging.2013.10.081, 24216223 PMC3880636

[bib15] Buckley, R. F., Schultz, A. P., Hedden, T., Papp, K. V., Hanseeuw, B. J., Marshall, G., … Chhatwal, J. P. (2017). Functional network integrity presages cognitive decline in preclinical Alzheimer disease. Neurology, 89(1), 29–37. 10.1212/WNL.0000000000004059, 28592457 PMC5496516

[bib16] Buckner, R. L., Sepulcre, J., Talukdar, T., Krienen, F. M., Liu, H., Hedden, T., … Johnson, K. A. (2009). Cortical hubs revealed by intrinsic functional connectivity: Mapping, assessment of stability, and relation to Alzheimer’s disease. Journal of Neuroscience, 29(6), 1860–1873. 10.1523/JNEUROSCI.5062-08.2009, 19211893 PMC2750039

[bib17] Bullmore, E., & Sporns, O. (2012). The economy of brain network organization. Nature Reviews Neuroscience, 13(5), 336–349. 10.1038/nrn3214, 22498897

[bib18] Cao, R., Wang, X., Gao, Y., Li, T., Zhang, H., Hussain, W., … Xiang, J. (2020). Abnormal anatomical rich-club organization and structural–functional coupling in mild cognitive impairment and Alzheimer’s disease. Frontiers in Neurology, 11, 53. 10.3389/fneur.2020.00053, 32117016 PMC7013042

[bib19] Chen, A. A., Beer, J. C., Tustison, N. J., Cook, P. A., Shinohara, R. T., Shou, H., & Alzheimer’s Disease Neuroimaging Initiative. (2022). Mitigating site effects in covariance for machine learning in neuroimaging data. Human Brain Mapping, 43(4), 1179–1195. 10.1002/hbm.25688, 34904312 PMC8837590

[bib20] Chhatwal, J. P., Schultz, A. P., Johnson, K., Benzinger, T. L. S., Jack, C., Jr., Ances, B. M., … Sperling, R. A. (2013). Impaired default network functional connectivity in autosomal dominant Alzheimer disease. Neurology, 81(8), 736–744. 10.1212/WNL.0b013e3182a1aafe, 23884042 PMC3776464

[bib21] Cirrito, J. R., Yamada, K. A., Finn, M. B., Sloviter, R. S., Bales, K. R., May, P. C., … Holtzman, D. M. (2005). Synaptic activity regulates interstitial fluid amyloid-β levels in vivo. Neuron, 48(6), 913–922. 10.1016/j.neuron.2005.10.028, 16364896

[bib22] Cole, M. W., Pathak, S., & Schneider, W. (2010). Identifying the brain’s most globally connected regions. NeuroImage, 49(4), 3132–3148. 10.1016/j.neuroimage.2009.11.001, 19909818

[bib23] Cope, T. E., Rittman, T., Borchert, R. J., Jones, P. S., Vatansever, D., Allinson, K., … Rowe, J. B. (2018). Tau burden and the functional connectome in Alzheimer’s disease and progressive supranuclear palsy. Brain, 141(2), 550–567. 10.1093/brain/awx347, 29293892 PMC5837359

[bib24] Crossley, N. A., Mechelli, A., Scott, J., Carletti, F., Fox, P. T., McGuire, P., & Bullmore, E. T. (2014). The hubs of the human connectome are generally implicated in the anatomy of brain disorders. Brain, 137(8), 2382–2395. 10.1093/brain/awu132, 25057133 PMC4107735

[bib25] Dai, Z., Yan, C., Li, K., Wang, Z., Wang, J., Cao, M., … He, Y. (2015). Identifying and mapping connectivity patterns of brain network hubs in Alzheimer’s disease. Cerebral Cortex, 25(10), 3723–3742. 10.1093/cercor/bhu246, 25331602

[bib26] Daianu, M., Jahanshad, N., Nir, T. M., Jack, C. R., Jr., Weiner, M. W., Bernstein, M. A., … Alzheimer's Disease Neuroimaging Initiative. (2015). Rich club analysis in the Alzheimer’s disease connectome reveals a relatively undisturbed structural core network. Human Brain Mapping, 36(8), 3087–3103. 10.1002/hbm.22830, 26037224 PMC4504816

[bib27] delEtoile, J., & Adeli, H. (2017). Graph theory and brain connectivity in Alzheimer’s disease. Neuroscientist, 23(6), 616–626. 10.1177/1073858417702621, 28406055

[bib28] Dennis, E. L., & Thompson, P. M. (2014). Functional brain connectivity using fMRI in aging and Alzheimer’s disease. Neuropsychology Review, 24(1), 49–62. 10.1007/s11065-014-9249-6, 24562737 PMC4109887

[bib29] de Haan, W., Mott, K., van Straaten, E. C. W., Scheltens, P., & Stam, C. J. (2012). Activity dependent degeneration explains hub vulnerability in Alzheimer’s disease. PLOS Computational Biology, 8(8), e1002582. 10.1371/journal.pcbi.1002582, 22915996 PMC3420961

[bib30] de Haan, W., van Straaten, E. C. W., Gouw, A. A., & Stam, C. J. (2017). Altering neuronal excitability to preserve network connectivity in a computational model of Alzheimer’s disease. PLOS Computational Biology, 13(9), e1005707. 10.1371/journal.pcbi.1005707, 28938009 PMC5627940

[bib31] de Reus, M. A., & van den Heuvel, M. P. (2013). Rich club organization and intermodule communication in the cat connectome. Journal of Neuroscience, 33(32), 12929–12939. 10.1523/JNEUROSCI.1448-13.2013, 23926249 PMC6619725

[bib32] Dickerson, B. C., & Sperling, R. A. (2005). Neuroimaging biomarkers for clinical trials of disease-modifying therapies in Alzheimer’s disease. NeuroRx, 2(2), 348–360. 10.1602/neurorx.2.2.348, 15897955 PMC1064996

[bib33] Diez, I., & Sepulcre, J. (2021). Unveiling the neuroimaging-genetic intersections in the human brain. Current Opinion in Neurology, 34(4), 480–487. 10.1097/WCO.0000000000000952, 34227572 PMC8265485

[bib34] Drakesmith, M., Caeyenberghs, K., Dutt, A., Lewis, G., David, A. S., & Jones, D. K. (2015). Overcoming the effects of false positives and threshold bias in graph theoretical analyses of neuroimaging data. NeuroImage, 118, 313–333. 10.1016/j.neuroimage.2015.05.011, 25982515 PMC4558463

[bib35] Drzezga, A., Becker, J. A., Van Dijk, K. R. A., Sreenivasan, A., Talukdar, T., Sullivan, C., … Sperling, R. A. (2011). Neuronal dysfunction and disconnection of cortical hubs in non-demented subjects with elevated amyloid burden. Brain, 134(Pt 6), 1635–1646. 10.1093/brain/awr066, 21490054 PMC3102239

[bib36] Fabiani, M., Gordon, B. A., Maclin, E. L., Pearson, M. A., Brumback-Peltz, C. R., Low, K. A., … Gratton, G. (2014). Neurovascular coupling in normal aging: A combined optical, ERP and fMRI study. NeuroImage, 85, 592–607. 10.1016/j.neuroimage.2013.04.113, 23664952 PMC3791333

[bib37] Fischl, B. (2012). FreeSurfer. NeuroImage, 62(2), 774–781. 10.1016/j.neuroimage.2012.01.021, 22248573 PMC3685476

[bib38] Fornito, A., Zalesky, A., Bassett, D. S., Meunier, D., Ellison-Wright, I., Yücel, M., … Bullmore, E. T. (2011). Genetic influences on cost-efficient organization of human cortical functional networks. Journal of Neuroscience, 31(9), 3261–3270. 10.1523/JNEUROSCI.4858-10.2011, 21368038 PMC6623940

[bib39] Franzmeier, N., Neitzel, J., Rubinski, A., Smith, R., Strandberg, O., Ossenkoppele, R., … Alzheimer’s Disease Neuroimaging Initiative (ADNI). (2020). Functional brain architecture is associated with the rate of tau accumulation in Alzheimer’s disease. Nature Communications, 11(1), 347. 10.1038/s41467-019-14159-1, 31953405 PMC6969065

[bib40] Frontzkowski, L., Ewers, M., Brendel, M., Biel, D., Ossenkoppele, R., Hager, P., … Franzmeier, N. (2022). Earlier Alzheimer’s disease onset is associated with tau pathology in brain hub regions and facilitated tau spreading. Nature Communications, 13(1), 4899. 10.1038/s41467-022-32592-7, 35987901 PMC9392750

[bib41] Garrison, K. A., Scheinost, D., Finn, E. S., Shen, X., & Constable, R. T. (2015). The (in)stability of functional brain network measures across thresholds. NeuroImage, 118, 651–661. 10.1016/j.neuroimage.2015.05.046, 26021218 PMC4554838

[bib42] Gordon, B. A., Blazey, T. M., Su, Y., Hari-Raj, A., Dincer, A., Flores, S., … Benzinger, T. L. S. (2018). Spatial patterns of neuroimaging biomarker change in individuals from families with autosomal dominant Alzheimer’s disease: A longitudinal study. Lancet Neurology, 17(3), 241–250. 10.1016/S1474-4422(18)30028-0, 29397305 PMC5816717

[bib43] Gratton, C., Nomura, E. M., Pérez, F., & D’Esposito, M. (2012). Focal brain lesions to critical locations cause widespread disruption of the modular organization of the brain. Journal of Cognitive Neuroscience, 24(6), 1275–1285. 10.1162/jocn_a_00222, 22401285 PMC3575518

[bib44] Guimerà, R., & Nunes Amaral, L. A. (2005). Functional cartography of complex metabolic networks. Nature, 433(7028), 895–900. 10.1038/nature03288, 15729348 PMC2175124

[bib45] Hagmann, P., Cammoun, L., Gigandet, X., Meuli, R., Honey, C. J., Wedeen, V. J., & Sporns, O. (2008). Mapping the structural core of human cerebral cortex. PLOS Biology, 6(7), e159. 10.1371/journal.pbio.0060159, 18597554 PMC2443193

[bib46] Hampel, H., Hardy, J., Blennow, K., Chen, C., Perry, G., Kim, S. H., … Vergallo, A. (2021). The amyloid-β pathway in Alzheimer’s disease. Molecular Psychiatry, 26(10), 5481–5503. 10.1038/s41380-021-01249-0, 34456336 PMC8758495

[bib47] Honey, C. J., & Sporns, O. (2008). Dynamical consequences of lesions in cortical networks. Human Brain Mapping, 29(7), 802–809. 10.1002/hbm.20579, 18438885 PMC6870962

[bib48] Jack, C. R., Jr., Knopman, D. S., Jagust, W. J., Shaw, L. M., Aisen, P. S., Weiner, M. W., … Trojanowski, J. Q. (2010). Hypothetical model of dynamic biomarkers of the Alzheimer’s pathological cascade. Lancet Neurology, 9(1), 119–128. 10.1016/S1474-4422(09)70299-6, 20083042 PMC2819840

[bib49] Jenkinson, M., Beckmann, C. F., Behrens, T. E. J., Woolrich, M. W., & Smith, S. M. (2012). FSL. NeuroImage, 62(2), 782–790. 10.1016/j.neuroimage.2011.09.015, 21979382

[bib50] Jones, D. T., Graff-Radford, J., Lowe, V. J., Wiste, H. J., Gunter, J. L., Senjem, M. L., … Jack, C. R. (2017). Tau, amyloid, and cascading network failure across the Alzheimer’s disease spectrum. Cortex, 97, 143–159. 10.1016/j.cortex.2017.09.018, 29102243 PMC5773067

[bib51] Jones, D. T., Knopman, D. S., Gunter, J. L., Graff-Radford, J., Vemuri, P., Boeve, B. F., … on behalf of the Alzheimer’s Disease Neuroimaging Initiative. (2016). Cascading network failure across the Alzheimer’s disease spectrum. Brain, 139(2), 547–562. 10.1093/brain/awv338, 26586695 PMC4805086

[bib52] Jones, D. T., Machulda, M. M., Vemuri, P., McDade, E. M., Zeng, G., Senjem, M. L., … Jack, C. R., Jr. (2011). Age-related changes in the default mode network are more advanced in Alzheimer disease. Neurology, 77(16), 1524–1531. 10.1212/WNL.0b013e318233b33d, 21975202 PMC3198977

[bib53] Knol, M. J., Pestman, W. R., & Grobbee, D. E. (2011). The (mis)use of overlap of confidence intervals to assess effect modification. European Journal of Epidemiology, 26(4), 253–254. 10.1007/s10654-011-9563-8, 21424218 PMC3088813

[bib54] Koch, G., Casula, E. P., Bonnì, S., Borghi, I., Assogna, M., Minei, M., … Martorana, A. (2022). Precuneus magnetic stimulation for Alzheimer’s disease: A randomized, sham-controlled trial. Brain, 145(11), 3776–3786. 10.1093/brain/awac285, 36281767 PMC9679166

[bib55] Kotlarz, P., Nino, J. C., & Febo, M. (2022). Connectomic analysis of Alzheimer’s disease using percolation theory. Network Neuroscience, 6(1), 213–233. 10.1162/netn_a_00221, 36605889 PMC9810282

[bib56] Liang, X., Zou, Q., He, Y., & Yang, Y. (2013). Coupling of functional connectivity and regional cerebral blood flow reveals a physiological basis for network hubs of the human brain. Proceedings of the National Academy of Sciences, 110(5), 1929–1934. 10.1073/pnas.1214900110, 23319644 PMC3562840

[bib57] Liu, G., Shen, C., & Qiu, A. (2023). Amyloid-β accumulation in relation to functional connectivity in aging: A longitudinal study. NeuroImage, 275, 120146. 10.1016/j.neuroimage.2023.120146, 37127190

[bib58] Mattson, M. P., Cheng, B., Davis, D., Bryant, K., Lieberburg, I., & Rydel, R. E. (1992). Beta-amyloid peptides destabilize calcium homeostasis and render human cortical neurons vulnerable to excitotoxicity. Journal of Neuroscience, 12(2), 376–389. 10.1523/JNEUROSCI.12-02-00376.1992, 1346802 PMC6575616

[bib151] Mazumder, R., & Hastie, T. (2012). The graphical lasso: New insights and alternatives. Electronic Journal of Statistics, 6, 2125–2149. 10.1214/12-EJS740, 25558297 PMC4281944

[bib59] McKay, N. S., Gordon, B. A., Hornbeck, R. C., Dincer, A., Flores, S., Keefe, S. J., … Dominantly Inherited Alzheimer Network. (2023). Positron emission tomography and magnetic resonance imaging methods and datasets within the Dominantly Inherited Alzheimer Network (DIAN). Nature Neuroscience, 26(8), 1449–1460. 10.1038/s41593-023-01359-8, 37429916 PMC10400428

[bib152] Morris, J. C. (1993). The Clinical Dementia Rating (CDR): Current version and scoring rules. Neurology, 43(11), 2412–2414. 10.1212/WNL.43.11.2412-a, 8232972

[bib60] Musiek, E. S., & Holtzman, D. M. (2015). Three dimensions of the amyloid hypothesis: Time, space and “wingmen.” Nature Neuroscience, 18(6), 800–806. 10.1038/nn.4018, 26007213 PMC4445458

[bib61] Myers, N., Pasquini, L., Göttler, J., Grimmer, T., Koch, K., Ortner, M., … Sorg, C. (2014). Within-patient correspondence of amyloid-β and intrinsic network connectivity in Alzheimer’s disease. Brain, 137(7), 2052–2064. 10.1093/brain/awu103, 24771519 PMC4065018

[bib62] Palmqvist, S., Schöll, M., Strandberg, O., Mattsson, N., Stomrud, E., Zetterberg, H., … Hansson, O. (2017). Earliest accumulation of β-amyloid occurs within the default-mode network and concurrently affects brain connectivity. Nature Communications, 8(1), 1214. 10.1038/s41467-017-01150-x, 29089479 PMC5663717

[bib63] Payton, M. E., Greenstone, M. H., & Schenker, N. (2003). Overlapping confidence intervals or standard error intervals: What do they mean in terms of statistical significance? Journal of Insect Science, 3(1), 34. 10.1093/jis/3.1.34, 15841249 PMC524673

[bib64] Power, J. D., Cohen, A. L., Nelson, S. M., Wig, G. S., Barnes, K. A., Church, J. A., … Petersen, S. E. (2011). Functional network organization of the human brain. Neuron, 72(4), 665–678. 10.1016/j.neuron.2011.09.006, 22099467 PMC3222858

[bib65] Power, J. D., Mitra, A., Laumann, T. O., Snyder, A. Z., Schlaggar, B. L., & Petersen, S. E. (2014). Methods to detect, characterize, and remove motion artifact in resting state fMRI. NeuroImage, 84, 320–341. 10.1016/j.neuroimage.2013.08.048, 23994314 PMC3849338

[bib66] Power, J. D., Schlaggar, B. L., Lessov-Schlaggar, C. N., & Petersen, S. E. (2013). Evidence for hubs in human functional brain networks. Neuron, 79(4), 798–813. 10.1016/j.neuron.2013.07.035, 23972601 PMC3838673

[bib67] Raut, R. V., Mitra, A., Snyder, A. Z., & Raichle, M. E. (2019). On time delay estimation and sampling error in resting-state fMRI. NeuroImage, 194, 211–227. 10.1016/j.neuroimage.2019.03.020, 30902641 PMC6559238

[bib68] Rubinov, M., McIntosh, A. R., Valenzuela, M. J., & Breakspear, M. (2009). Simulation of neuronal death and network recovery in a computational model of distributed cortical activity. American Journal of Geriatric Psychiatry, 17(3), 210–217. 10.1097/JGP.0b013e318187137a, 19001355

[bib69] Rubinov, M., & Sporns, O. (2010). Complex network measures of brain connectivity: Uses and interpretations. NeuroImage, 52(3), 1059–1069. 10.1016/j.neuroimage.2009.10.003, 19819337

[bib70] Rubinov, M., & Sporns, O. (2011). Weight-conserving characterization of complex functional brain networks. NeuroImage, 56(4), 2068–2079. 10.1016/j.neuroimage.2011.03.069, 21459148

[bib71] Seitzman, B. A., Gratton, C., Marek, S., Raut, R. V., Dosenbach, N. U. F., Schlaggar, B. L., … Greene, D. J. (2020). A set of functionally-defined brain regions with improved representation of the subcortex and cerebellum. NeuroImage, 206, 116290. 10.1016/j.neuroimage.2019.116290, 31634545 PMC6981071

[bib72] Sheline, Y. I., & Raichle, M. E. (2013). Resting state functional connectivity in preclinical Alzheimer’s disease. Biological Psychiatry, 74(5), 340–347. 10.1016/j.biopsych.2012.11.028, 23290495 PMC3537262

[bib73] Smith, R. X., Strain, J. F., Tanenbaum, A., Fagan, A. M., Hassenstab, J., McDade, E., … Ances, B. M. (2021). Resting-state functional connectivity disruption as a pathological biomarker in autosomal dominant Alzheimer disease. Brain Connectivity, 11(3), 239–249. 10.1089/brain.2020.0808, 33430685 PMC8182476

[bib74] Song, J., Nair, V. A., Gaggl, W., & Prabhakaran, V. (2015). Disrupted brain functional organization in epilepsy revealed by graph theory analysis. Brain Connectivity, 5(5), 276–283. 10.1089/brain.2014.0308, 25647011 PMC4490776

[bib75] Sorg, C., Riedl, V., Perneczky, R., Kurz, A., & Wohlschläger, A. M. (2009). Impact of Alzheimer’s disease on the functional connectivity of spontaneous brain activity. Current Alzheimer Research, 6(6), 541–553. 10.2174/156720509790147106, 19747154

[bib76] Sperling, R. A., Aisen, P. S., Beckett, L. A., Bennett, D. A., Craft, S., Fagan, A. M., … Phelps, C. H. (2011). Toward defining the preclinical stages of Alzheimer’s disease: Recommendations from the National Institute on Aging-Alzheimer’s Association workgroups on diagnostic guidelines for Alzheimer’s disease. Alzheimer’s & Dementia, 7(3), 280–292. 10.1016/j.jalz.2011.03.003, 21514248 PMC3220946

[bib77] Sporns, O., Honey, C. J., & Kötter, R. (2007). Identification and classification of hubs in brain networks. PLOS ONE, 2(10), e1049. 10.1371/journal.pone.0001049, 17940613 PMC2013941

[bib78] Stam, C. J., de Haan, W., Daffertshofer, A., Jones, B. F., Manshanden, I., van Cappellen van Walsum, A. M., … Scheltens, P. (2009). Graph theoretical analysis of magnetoencephalographic functional connectivity in Alzheimer’s disease. Brain, 132(1), 213–224. 10.1093/brain/awn262, 18952674

[bib79] Strain, J. F., Brier, M. R., Tanenbaum, A., Gordon, B. A., McCarthy, J. E., Dincer, A., … for the Dominantly Inherited Alzheimer Network. (2022). Covariance-based vs. correlation-based functional connectivity dissociates healthy aging from Alzheimer disease. NeuroImage, 261, 119511. 10.1016/j.neuroimage.2022.119511, 35914670 PMC9750733

[bib80] Su, Y., D’Angelo, G. M., Vlassenko, A. G., Zhou, G., Snyder, A. Z., Marcus, D. S., … Benzinger, T. L. S. (2013). Quantitative analysis of PiB-PET with FreeSurfer ROIs. PLOS ONE, 8(11), e73377. 10.1371/journal.pone.0073377, 24223109 PMC3819320

[bib81] Termenon, M., Achard, S., Jaillard, A., & Delon-Martin, C. (2016). The “hub disruption index,” a reliable index sensitive to the brain networks reorganization. A study of the contralesional hemisphere in stroke. Frontiers in Computational Neuroscience, 10, 84. 10.3389/fncom.2016.00084, 27582702 PMC4987351

[bib82] Thomas, J. B., Brier, M. R., Bateman, R. J., Snyder, A. Z., Benzinger, T. L., Xiong, C., … Ances, B. M. (2014). Functional connectivity in autosomal dominant and late-onset Alzheimer disease. JAMA Neurology, 71(9), 1111–1122. 10.1001/jamaneurol.2014.1654, 25069482 PMC4240274

[bib83] Tomasi, D., Wang, G.-J., & Volkow, N. D. (2013). Energetic cost of brain functional connectivity. Proceedings of the National Academy of Sciences, 110(33), 13642–13647. 10.1073/pnas.1303346110, 23898179 PMC3746878

[bib84] Tu, W., Ma, Z., & Zhang, N. (2021). Brain network reorganization after targeted attack at a hub region. NeuroImage, 237, 118219. 10.1016/j.neuroimage.2021.118219, 34052466 PMC8289586

[bib85] Vaishnavi, S. N., Vlassenko, A. G., Rundle, M. M., Snyder, A. Z., Mintun, M. A., & Raichle, M. E. (2010). Regional aerobic glycolysis in the human brain. Proceedings of the National Academy of Sciences, 107(41), 17757–17762. 10.1073/pnas.1010459107, 20837536 PMC2955101

[bib86] van den Heuvel, M. P., & Sporns, O. (2013). Network hubs in the human brain. Trends in Cognitive Sciences, 17(12), 683–696. 10.1016/j.tics.2013.09.012, 24231140

[bib87] van Wijk, B. C. M., Stam, C. J., & Daffertshofer, A. (2010). Comparing brain networks of different size and connectivity density using graph theory. PLOS ONE, 5(10), e13701. 10.1371/journal.pone.0013701, 21060892 PMC2965659

[bib88] Váša, F., & Mišić, B. (2022). Null models in network neuroscience. Nature Reviews Neuroscience, 23(8), 493–504. 10.1038/s41583-022-00601-9, 35641793

[bib89] Vatansever, D., Schröter, M., Adapa, R. M., Bullmore, E. T., Menon, D. K., & Stamatakis, E. A. (2020). Reorganisation of brain hubs across altered states of consciousness. Scientific Reports, 10(1), 3402. 10.1038/s41598-020-60258-1, 32099008 PMC7042369

[bib90] Villain, N., Chételat, G., Grassiot, B., Bourgeat, P., Jones, G., Ellis, K. A., … the AIBL Research Group. (2012). Regional dynamics of amyloid-β deposition in healthy elderly, mild cognitive impairment and Alzheimer’s disease: A voxelwise PiB–PET longitudinal study. Brain, 135(7), 2126–2139. 10.1093/brain/aws125, 22628162

[bib91] Wagstyl, K., Sophie, A., Seidlitz, J., Vandekar, S., Mallard, T. T., Dear, R., … Raznahan, A. (2024). Transcriptional cartography integrates multiscale biology of the human cortex. eLife, 12, RP86933. 10.7554/eLife.86933, 38324465 PMC10945526

[bib92] Wang, G., Berry, S., Xiong, C., Hassenstab, J., Quintana, M., McDade, E. M., … Dominantly Inherited Alzheimer Network Trials Unit. (2018). A novel cognitive disease progression model for clinical trials in autosomal-dominant Alzheimer’s disease. Statistics in Medicine, 37(21), 3047–3055. 10.1002/sim.7811, 29761523 PMC6105413

[bib93] Wheelock, M. D., Strain, J. F., Mansfield, P., Tu, J. C., Tanenbaum, A., Preische, O., … Dominantly Inherited Alzheimer Network. (2023). Brain network decoupling with increased serum neurofilament and reduced cognitive function in Alzheimer’s disease. Brain, 146(7), 2928–2943. 10.1093/brain/awac498, 36625756 PMC10316768

[bib94] White, R. L., III, Campbell, M. C., Yang, D., Shannon, W., Snyder, A. Z., & Perlmutter, J. S. (2020). Little change in functional brain networks following acute levodopa in drug-naïve Parkinson’s disease. Movement Disorders, 35(3), 499–503. 10.1002/mds.27942, 31854465 PMC7138409

[bib95] Wiepert, D. A., Lowe, V. J., Knopman, D. S., Boeve, B. F., Graff-Radford, J., Petersen, R. C., … Jones, D. T. (2017). A robust biomarker of large-scale network failure in Alzheimer’s disease. Alzheimer’s & Dementia: Diagnosis, Assessment & Disease Monitoring, 6, 152–161. 10.1016/j.dadm.2017.01.004, 28275697 PMC5328758

[bib96] Wig, G. S. (2017). Segregated systems of human brain networks. Trends in Cognitive Sciences, 21(12), 981–996. 10.1016/j.tics.2017.09.006, 29100737

[bib97] Wisch, J. K., Roe, C. M., Babulal, G. M., Schindler, S. E., Fagan, A. M., Benzinger, T. L., … Ances, B. M. (2020). Resting state functional connectivity signature differentiates cognitively normal from individuals who convert to symptomatic Alzheimer’s disease. Journal of Alzheimer’s Disease, 74(4), 1085–1095. 10.3233/JAD-191039, 32144983 PMC7183885

[bib98] Xia, M., Wang, J., & He, Y. (2013). BrainNet viewer: A network visualization tool for human brain connectomics. PLOS ONE, 8(7), e68910. 10.1371/journal.pone.0068910, 23861951 PMC3701683

[bib99] Yu, M., Engels, M. M. A., Hillebrand, A., van Straaten, E. C. W., Gouw, A. A., Teunissen, C., … Stam, C. J. (2017). Selective impairment of hippocampus and posterior hub areas in Alzheimer’s disease: An MEG-based multiplex network study. Brain, 140(5), 1466–1485. 10.1093/brain/awx050, 28334883

[bib100] Yu, M., Sporns, O., & Saykin, A. J. (2021). The human connectome in Alzheimer disease—Relationship to biomarkers and genetics. Nature Reviews Neurology, 17(9), 545–563. 10.1038/s41582-021-00529-1, 34285392 PMC8403643

