# Erratum: Increasing hub disruption parallels dementia severity in autosomal dominant Alzheimer’s disease

**DOI:** 10.1162/netn_x_00462

**Published:** 2024-04-28

**Authors:** Jiaxin Cindy Tu, Peter R. Millar, Jeremy F. Strain, Andrew Eck, Babatunde Adeyemo, Abraham Z. Snyder, Alisha Daniels, Celeste Karch, Edward D. Huey, Eric McDade, Gregory S. Day, Igor Yakushev, Jason Hassenstab, John Morris, Jorge J. Llibre-Guerra, Laura Ibanez, Mathias Jucker, Patricio Chrem Mendez, Richard J. Perrin, Tammie L. S. Benzinger, Clifford R. Jack, Richard Betzel, Beau M. Ances, Adam T. Eggebrecht, Brian A. Gordon, Muriah D. Wheelock, the Dominantly Inherited Alzheimer Network

**Affiliations:** Department of Radiology, Washington University in St. Louis, St. Louis, MO, USA; Department of Neurology, Washington University in St. Louis, St. Louis, MO, USA; Department of Psychiatry, Washington University in St. Louis, St. Louis, MO, USA; Department of Psychiatry and Human Behavior, Warren Alpert Medical School of Brown University, Providence, RI, USA; Department of Neurology, Mayo Clinic, Jacksonville, FL, USA; Department of Nuclear Medicine, Technical University of Munich, Munich, Germany; NeuroGenomics and Informatics Center, Washington University in St. Louis, St. Louis, MO, USA; Department of Cellular Neurology, Hertie Institute for Clinical Brain Research, University of Tübingen, Tübingen, Germany; German Center for Neurodegenerative Diseases (DZNE), Tübingen, Germany; Memory Center, Fleni, Argentina; Department of Pathology and Immunology, Washington University in St. Louis, St. Louis, MO, USA; Department of Radiology, Mayo Clinic, Rochester, MN, USA; Department of Psychological and Brain Sciences, Indiana University, Bloomington, IN, USA

In the paper “Increasing hub disruption parallels dementia severity in autosomal dominant Alzheimer’s disease” published in *Network Neuroscience*, *8*(4), there are errors in the captions for the following figures:

Figure 2. Where it read “Mean (lower triangle) and standard deviation (upper triangle) of the Fisher *Z*-transformed FC matrix”, we have corrected it to “Mean of the Fisher *Z*-transformed FC matrix”.
Figure 3(A). “(right) cartoon illustrating that strength is calculated by summing the weights across the connected edges” was corrected to “(right) cartoon illustrating the definition of strength”.
Figure 4(A). “(middle) distribution of *within-strength* *Z*-score (*Z*) across the NC match 1 group” was corrected to “(middle) distribution of *within-module strength* *Z*-score (*Z*) across the NC match 1 group” and “Module centers are nodes with a high *Z-score*” was corrected to “Module centers are nodes with a high *Z*”.

None of the changes affect the results or conclusions.

