# Supplementary material for: Increasing hub disruption parallels dementia severity in autosomal dominant Alzheimer’s disease
Source: Netw Neurosci. 2024 Dec 10;8(4):1265–90. doi: 10.1162/netn_a_00395 (PMC11674321; doi:10.1162/netn_a_00395)
Supplement: Supplementary file 1 [file netn-8-4-1265-s001.pdf]

Supplementary Materials

Appendix A. Hub disruption calculated with the average Participation coefficient (Pc) and Within-module Z-Score (Z) as reference

Using the mean of the same young, healthy control (NC match 1) as a reference, we found a similar distribution of Pc and Z to previous literature (Power et al. 2013) (Figure4A; Supplementary Figure 15). We calculated the group-level hub disruption index with NC match 1 following the same process but with average Pc as the centrality measure (Figure 4B). We found that the reduction of functional connectivity is not disproportionally higher at connectors with high Pc (Table 2). This observation was also confirmed in the individual-level hub disruption index (Supplementary Figure 16), where a one-way ANOVAs showed no significant difference across the MC groups ($F(2,118) = 2.5$, $p = 0.09$, Figure 4C) or the NC groups ($F(2, 81) = 0.7$, $p = 0.51$; Figure 4D).

The group-level hub disruption index with Z in NC match 1 as the reference (Figure 4E) showed a qualitatively similar pattern to the results using the NC match 1 average global connectivity strength as a reference (Figure 3): all MC groups had a significant negative hub disruption index while the NC groups did not (FDR-adjusted $p < 0.05$) (Table 2). We found no significant differences in slopes between MC (CDR=0.5) and MC (CDR = 0) ($F(1,488) = 0.9$, $p = 0.33$, partial $\eta^2 = 0.0019$), or between MC (CDR=0.5) and MC (CDR≥1) ($F(1,488) = 2.8$, $p = 0.09$, partial $\eta^2 = 0.0058$). However, there was a significant difference in slope between MC (CDR=0) compared to MC (CDR ≥1) ($F(1,488) = 6.5$, $p = 0.01$, partial $\eta^2 = 0.0131$). This observation was also confirmed in the individual-level hub disruption index (Supplementary Figure 17). All MC groups have hub disruption as indexed with a regression slope smaller than 0 (FDR-adjusted $p < 0.05$): MC (CDR=0) (M = -5.0, SD = 10.3, Cohen's $d = -0.5$, $t(68) = -4.0$, $p < 0.001$); MC (CDR=0.5) (M = -7.2, SD = 10.6, Cohen's $d = -0.7$, $t(31) = -3.9$, $p = 0.001$); MC (CDR≥1) (M = -12.7, SD = 11.3, Cohen's $d = -1.1$, $t(19) = -5.0$, $p < 0.001$). A one-way ANOVA on the MC groups showed that there were significant differences in at least one of the pairs ($F(2,118) = 4.2$, $p = 0.02$) (Figure 4F). A post-hoc t-test showed that MC (CDR=0) and MC (CDR≥1) groups had significantly different hub disruption index (FDR-adjusted $p = 0.01$). In contrast, a one-way ANOVA on the NC groups showed no significant difference in any of the pairs ($F(2, 81) = 0.6$, $p = 0.54$) (Figure 4G). Moreover, we validated our group-level results at different edge density thresholds to show that our results are consistent across thresholds (Supplementary Figure 18).

Additionally, while less interpretable, hub disruption index could be calculated with the % Pc and Z difference instead of the functional connectivity strength as dependent variables. Since Z contains both positive and negative values, dividing by Z to calculate % difference is tricky. Therefore, we used within module strength (within S) instead of within-module strength Z-score (Z) as the dependent variable. For Pc, we found that hub disruption indices at all MC groups and NC match 3 were significantly below 0 (Supplementary Table 6, FDR-adjusted $p < 0.05$). We found significant differences in slopes between MC (CDR=0.5) and MC (CDR=0) ($F(1,488) = 87.953$, $p < 0.0001$, partial $\eta^2 = 0.1527$), or between MC (CDR=0.5) and MC (CDR≥1) ($F(1,488) = 118.79$, $p < 0.0001$, partial $\eta^2 = 0.1958$). In addition, we confirm our results using individual hub disruption indices in MC (Supplementary Figure 19B) and NC (Supplementary Figure 19C). All MC groups have hub disruption as indexed with a regression slope <0 (FDR-adjusted $p < 0.05$): MC (CDR=0) (M = -1.7, SD = 10.9, Cohen's $d = -0.2$, $t(68) = -1.3$, $p = 0.31$); MC (CDR=0.5) (M = -11.6, SD = 15.3, Cohen's $d = -0.8$, $t(31) = -4.3$, $p = 0.0005$); MC (CDR≥1) (M = -26.6, SD = 14.5, Cohen's $d = -1.8$, $t(19) = -8.2$, $p < 0.0001$). A one-way ANOVA on the MC groups showed that there were significant differences in at least one of the pairs ($F(2,118) = 30.7$, $p < 0.001$), and a post-hoc t-test showed that all groups are significantly different from each other (FDR-adjusted $p < 0.001$). For Z, we found that hub disruption indices at MC (CDR=0) and MC (CDR≥1) groups were significantly below 0 (Supplementary Table 6, FDR-adjusted $p < 0.05$). We found significant differences in slopes between MC (CDR=0.5) and MC (CDR=0) ($F(1,488) = 0.739$, $p = 0.391$, partial $\eta^2 = 0.0015$),

or between MC (CDR=0.5) and MC (CDR≥1) ($F(1,488) = 4.9958$, $p = 0.026$, partial $\eta^2 = 0.0101$). In addition, we confirm our results using individual hub disruption indices in MC (Supplementary Figure 19E) and NC (Supplementary Figure 19F). All MC groups have hub disruption as indexed with a regression slope smaller than 0 (FDR-adjusted $p < 0.05$): MC (CDR=0) (M = -1.7, SD = 10.9, Cohen's $d = -0.2$, $t(68) = -3.9$, $p = 0.0007$); MC (CDR=0.5) (M = -11.6, SD = 15.3, Cohen's $d = -0.8$, $t(31) = -2.4$, $p = 0.04$); MC (CDR≥1) (M = -26.6, SD = 14.5, Cohen's $d = -1.8$, $t(19) = -5.7$, $p = 0.0001$). A one-way ANOVA on the MC groups showed that there was no significant difference in at least one of the pairs ($F(2,118) = 2.4$, $p = 0.09$).

Supplementary Tables

Supplementary Table 1. Sample Characteristics (mutation carriers and non-carriers)

Measure	Non-Carrier (N = 85)	Mutation Carrier (N = 122)	Test statistic	d.f.	*p*-value
			χ^2		
Sex (M/F)	34/51	58/64	3.70	1	0.28
CDR (0, 0.5, ≥1) n (%)	85 (100%), 0 (%), 0 (%)	70 (57.38%), 32 (26.23%), 20 (16.39%)	48.38	2	<0.001
	median	median	Mann-Whitney U	z	*p*-value
Age (yrs)	37.9	40.2	8782	0.1368	0.8912
Education (yrs)	15	14	9643	1.9109	0.056
EYO	-8.9	-6.7	8504	0.7913	0.4287
CCS[a]	0.06	-0.81	1065	5.5011	<0.001

a Missing 10 participant.

Medians and Mann Whitney test statistic reported (Shapiro-Wilk test of normality $p < 0.001$)
EYO; estimated years from expected symptom onset; CCS, Cognitive Composite Score; CDR, Clinical Dementia Rating.

Supplementary Table 2. Sequence details

MP-RAGE	TR = 2300ms; TE = 2.95ms; flip angle 9˚; TI = 900ms; 256x240 acquisition matrix; 176 sagittal slices 1x1x1.2mm resolution.
fMRI	TR = 2200ms; TE = 27ms; flip angle = 90˚;64x64 acquisition matrix, 36 axial slices in ascending interleaved order; 4mm isotropic resolution.
	TR = 2200ms; TE = 30ms; flip angle = 80˚; 64x58 acquisition matrix; 36 axial slices in ascending interleaved order; 3.3mm isotropic resolution.
	TR = 3000ms; TE = 30ms; flip angle = 80˚; 64x58 acquisition matrix; 48 axial slices in ascending interleaved order; 3.3mm isotropic resolution.

Supplementary Table 3. ROI specification (available as a separate excel file attachment).

Supplementary Table 4. Tests for assessing general cognitive functions to give cognitive composite scores (Wang et al. 2018)

Test	Explained	Range
MINI MENTAL STATE EXAM(Folstein, Folstein, and McHugh 1975)	Scored according to the UDS guidebook.	0-30
WMS-R LOGICAL MEMORY IIA - DELAYED(Wechsler 1987)	Administered after WAIS-R Digit Symbol in prescribed UDS order, and scored according to WMS-R manual	0-25
WAIS-R DIGIT SYMBOL(Wechsler 1981)	This is an enlarged Digit Symbol form that measures 15 x 24 cm rather than 9.5 x 13 cm as in the standard WAIS-R. Otherwise administered and raw scored according to WAIS-R manual.	0-93
WORD LIST RECALL - Delayed	Number of words from word list recalled after delay interval.	0-16
Source: DIAN CODEBOOK V1.5 1_1_2019_PDF		

Supplementary Table 5. Comparing the distribution of individual hub disruption index to zero

**Groups**	Mean	S.D.	Cohen's d	d.f.	t	p
MC (CDR=0)	-5.6	10.9	-0.51	68	-4.28	<0.001
MC (CDR=0.5)	-9.6	9.6	-1.00	31	-5.64	<0.001
MC (CDR≥1)	-16.7	10.3	-1.62	19	-7.25	<0.001
NC (match 1)	0	13.9	0	51	0	1.0
NC (match 2)	-0.8	14.7	-0.06	16	-0.24	1.0
NC (match 3)	-1.3	9.1	-0.15	14	-0.57	1.0

Supplementary Table 6. Group-level hub disruption (using metrics in NC match1 as baseline) across CDR stages in MC and across age in NC (FDR-adjusted).

Participation Coefficient (Pc)					
	Group	κ_S	$F(1,244)$	p	R^2
MC	CDR=0	-1.7	6.83	0.012	0.03
	CDR=0.5	-11.6	192	<0.0001	0.44
	CDR≥1	-26.6	590	<0.0001	0.71
NC	match 2	-0.1	0.0262	0.872	0.00
	match 3	-5.9	42.9	<0.0001	0.15
Within-module Strength Z-score (Z)					
	Group	κ_S	$F(1,244)$	p	R^2
MC	CDR=0	-6.3	13.3	0.0008	0.05
	CDR=0.5	-4.1	4.32	0.0646	0.02
	CDR≥1	-11.4	18.5	<0.0001	0.07
NC	match 2	-3.6	2.39	0.154	0.01
	match 3	-3.1	1.73	0.190	0.01

Supplementary Table 7. Regression of hub disruption index on Cognitive Composite Score (CCS). Response: Cognitive Composite Scores (CCS). β, coefficient of regression. *** $p < 0.001$, ** $p < 0.01$, * $p < 0.05$. Random effect: family.
(κ_S derived using participation coefficient)

Edge threshold	β_κ_S	β_*Education*	β_*Age*	β_*Sex (Male)*	β_*FD*
5%	0.0002	0.10***	-0.06***	-0.16	-3.37
10%	0.01	0.10***	-0.06***	-0.20	-3.59
20%	-0.01	0.11***	-0.06***	-0.15	-3.06
30%	-0.01	0.11***	-0.06***	-0.15	-3.11
40%	-0.01	0.11***	-0.06***	-0.15	-3.13

Supplementary Table 8. Regression of hub disruption index on Cognitive Composite Score (CCS). Response: Cognitive Composite Scores (CCS). β, coefficient of regression. *** $p < 0.001$, ** $p < 0.01$, * $p < 0.05$. Random effect: family.
(κ_S derived using within-module Z)

Edge threshold	β_κ_S	β_*Education*	β_*Age*	β_*Sex (Male)*	β_*FD*
5%	0.02**	0.10***	-0.06***	-0.17	-3.19
10%	0.02*	0.10***	-0.06***	-0.17	-3.39
20%	0.04**	0.11***	-0.06***	-0.17	-3.57
30%	0.05*	0.11***	-0.06***	-0.17	-3.65
40%	0.07**	0.10***	-0.06***	-0.16	-3.68

Supplementary Table 9. AUC for detection of mutation carrier at different EYO stages

EYO Range (yrs)	[-40, -20]	[-20, -15]	[-15, -10]	[-10, -5]	[-5,0]	[0,5]	[5,30]
N	28	33	29	28	31	32	24
AUC	0.56	0.53	0.72	0.78	0.68	0.79	0.83

Supplementary Table 10. AUC for detection of mutation carrier at different age

Age Range (yrs)	[0,30]	[30,35]	[35,40]	[40,45]	[45,50]	[50,55]	[55,70]
N	41	35	31	30	23	21	24
AUC	0.60	0.61	0.67	0.64	0.86	0.79	0.87

Supplementary Figures

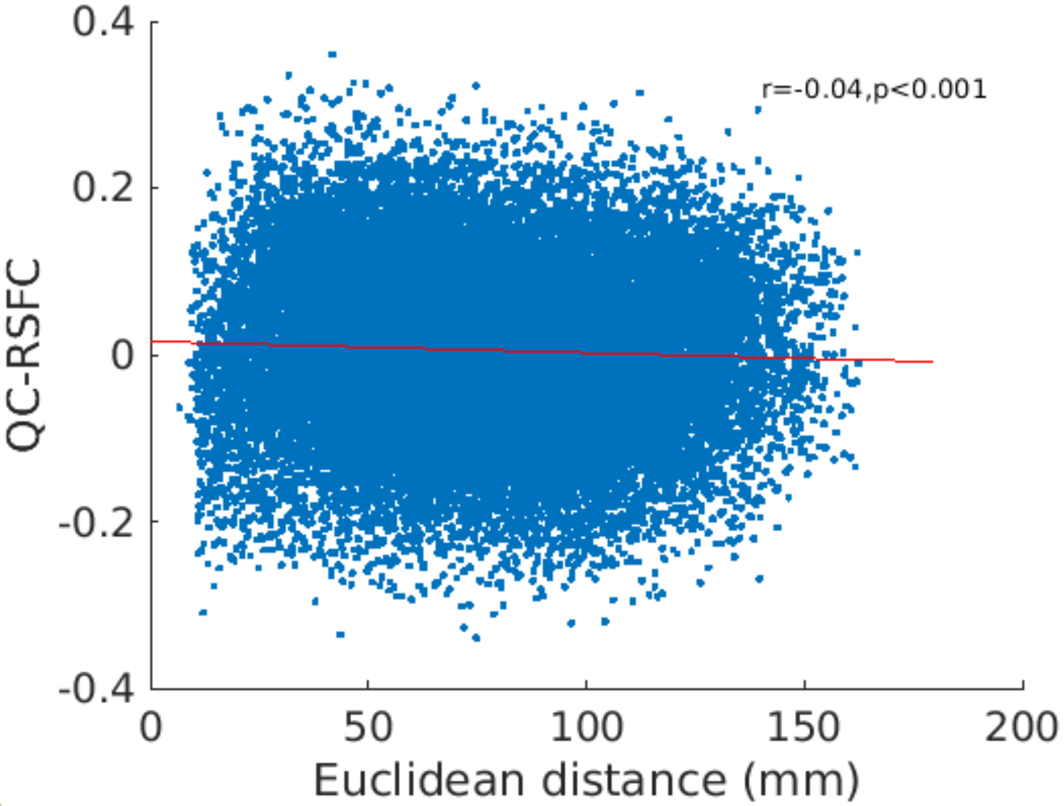

Supplementary Figure 1. **QC-RSFC correlation with Euclidean distance**. The y-axis shows the mean FD of retained frame correlation with RSFC at each edge across participants and the x-axis shows the Euclidean distance for the edges. The correlation r = -0.04, $p < 0.001$.

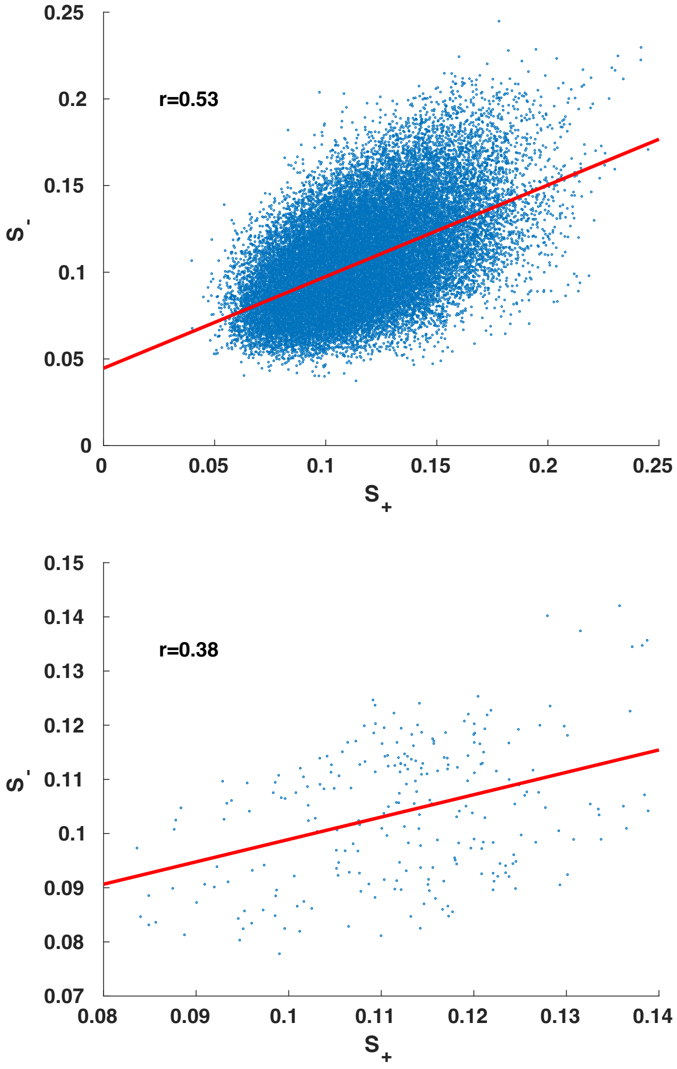

Supplementary Figure 2. Nodal strength (S) calculated with positive and negative connections in the full matrix without threshold are highly correlated. (Top) Across all subjects. Pearson's correlation, r = 0.53. (Bottom) Group average. Pearson's correlation, r = 0.38. S_+ and S_- are the average of positive weights and negative weights, respectively. Red line shows linear fit.

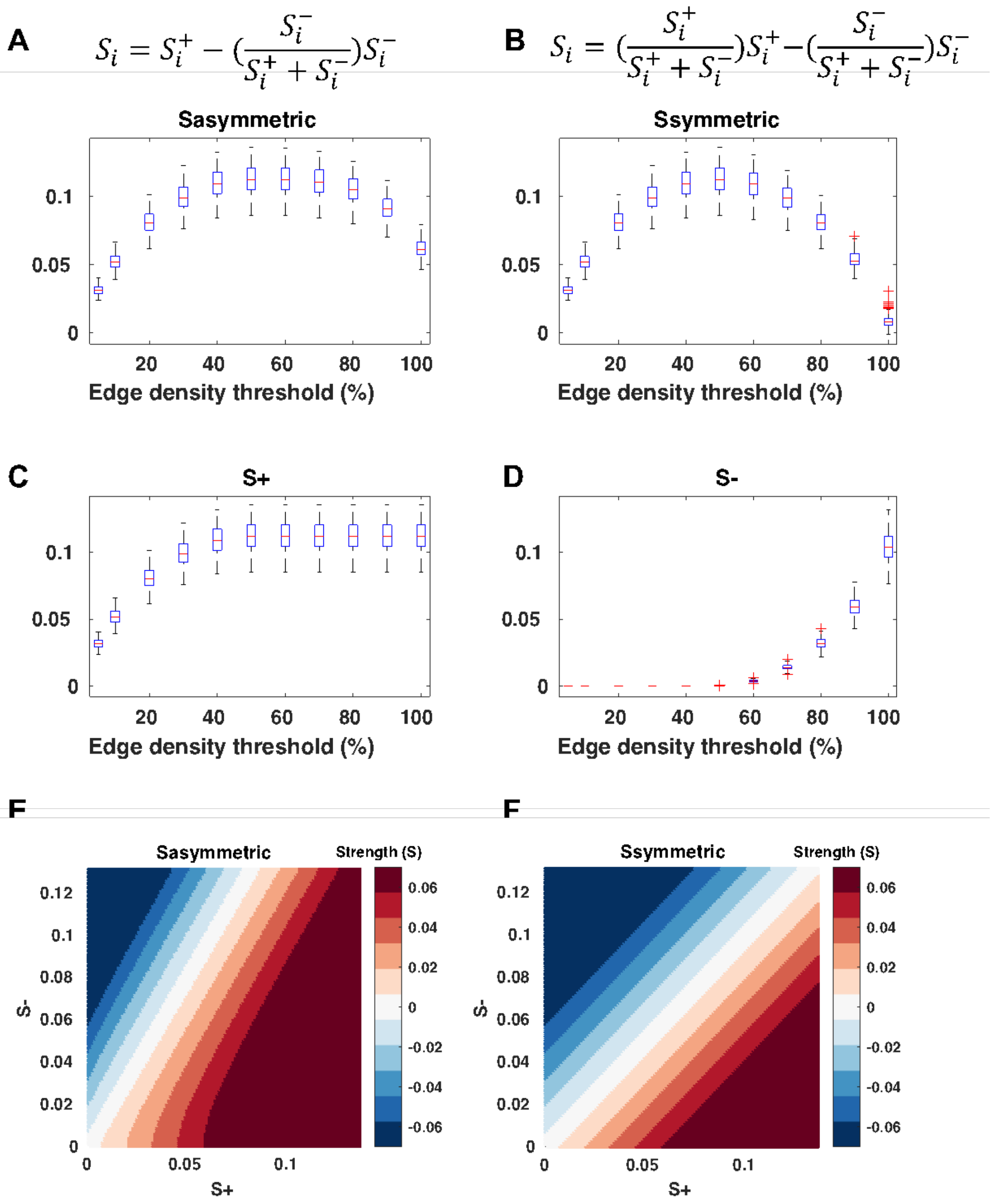

Supplementary Figure 3. The calculation of nodal strength from positive and negative strengths. a) Formula for asymmetric strength calculation and the asymmetric strength size at different densities. b) Formula for symmetric strength calculation and the symmetric strength size at different densities. c) Strength for positive edges at different densities. d) Strength for negative edges at different densities. e) Asymmetric strength for combinations of S+ and S-. f) Symmetric strength for combinations of S+ and S-.

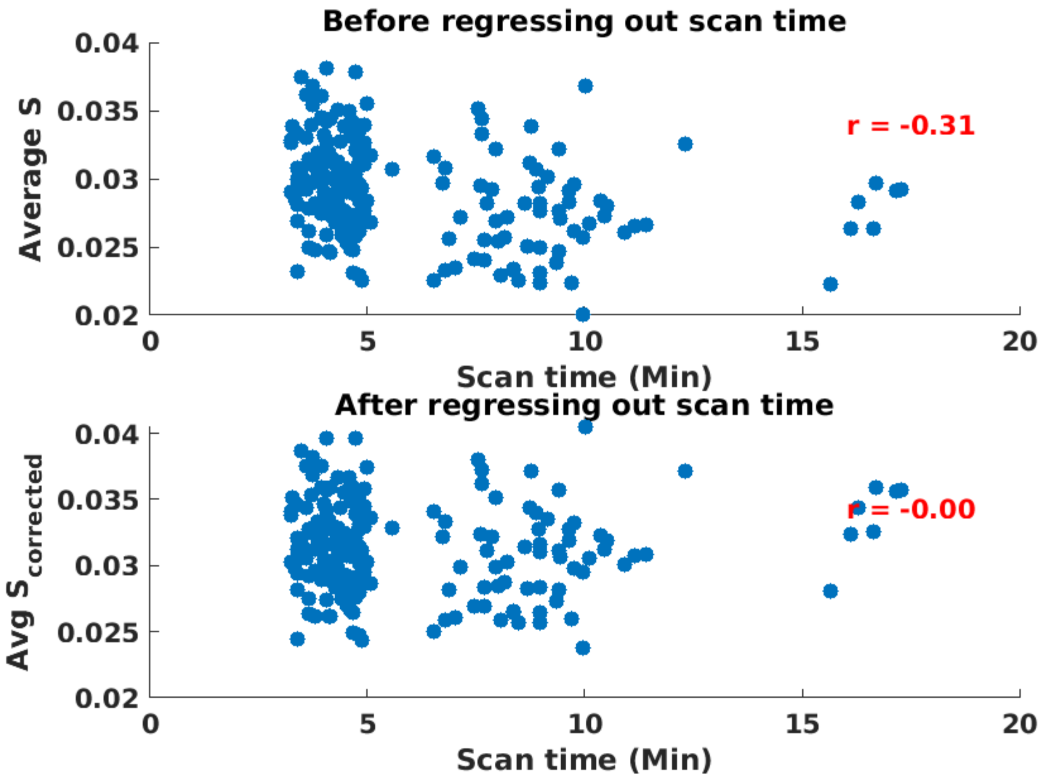

Supplementary Figure 4. Correlation between Average nodal strength (S) and retained minutes after frame censoring. (Top) before and (Bottom) after linear regression to correct for the correlation between average nodal strength and remaining scan time. Edge density = 5%.

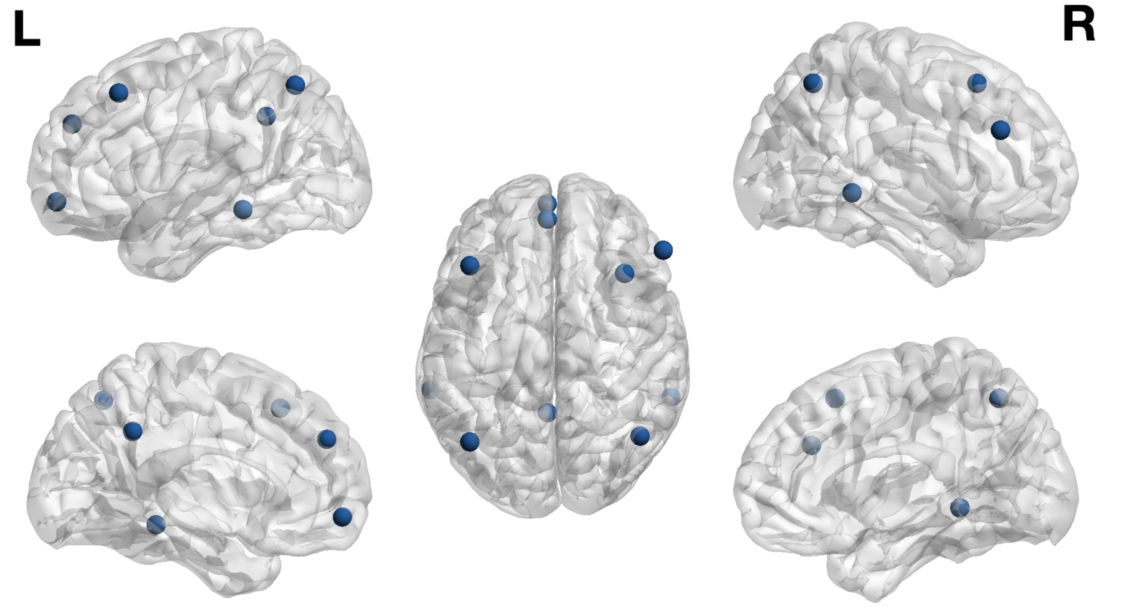

Supplementary Figure 5. Hubs identified in Buckner et al., 2009 J.Neurosci. Nodes generated with the MNI coordinates from the original paper.

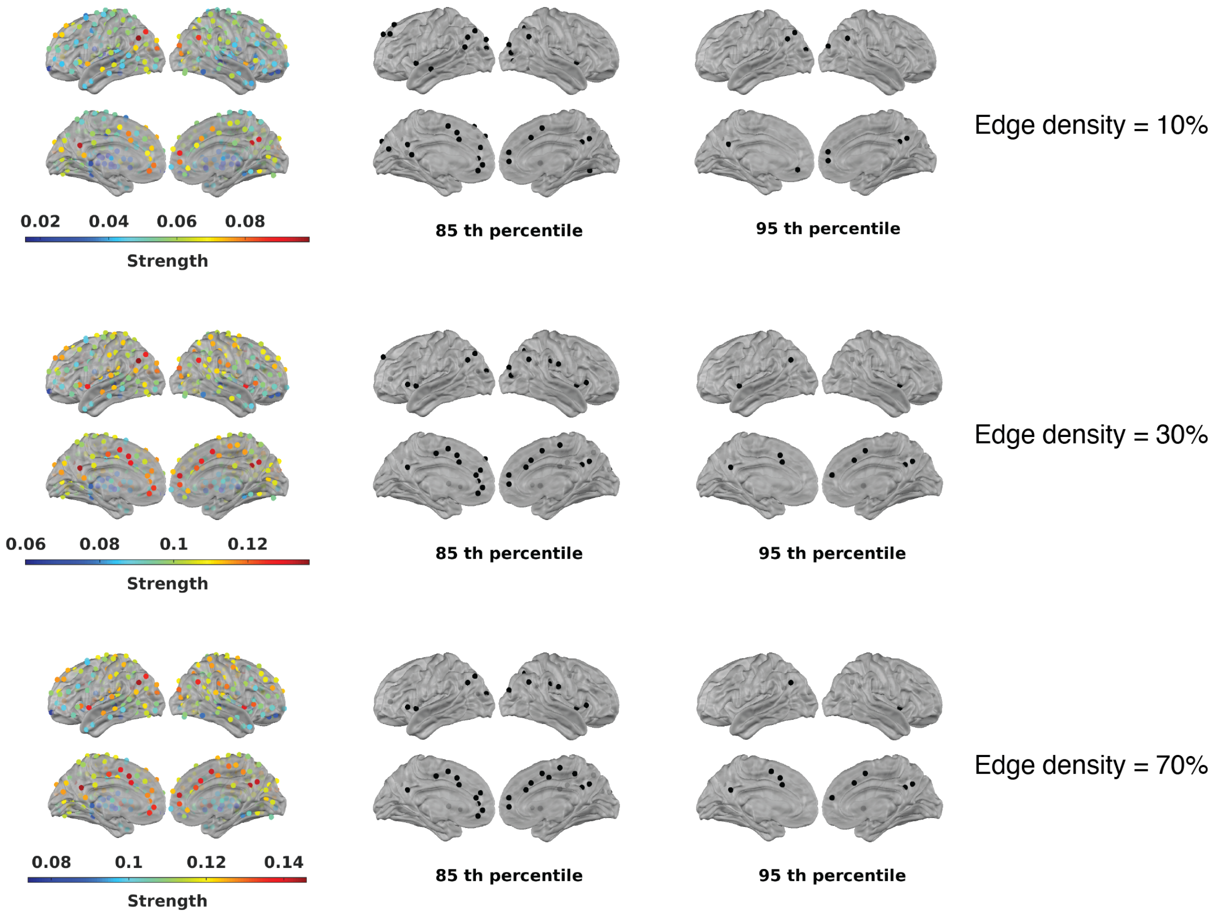

Supplementary Figure 6. Distribution of reference nodal strength and high strength hubs at different edge density thresholds. Edge density 10%, 30%, 70%. ROIs with strength higher than the 85th percentile and 95th percentile are shown in the middle and right column.

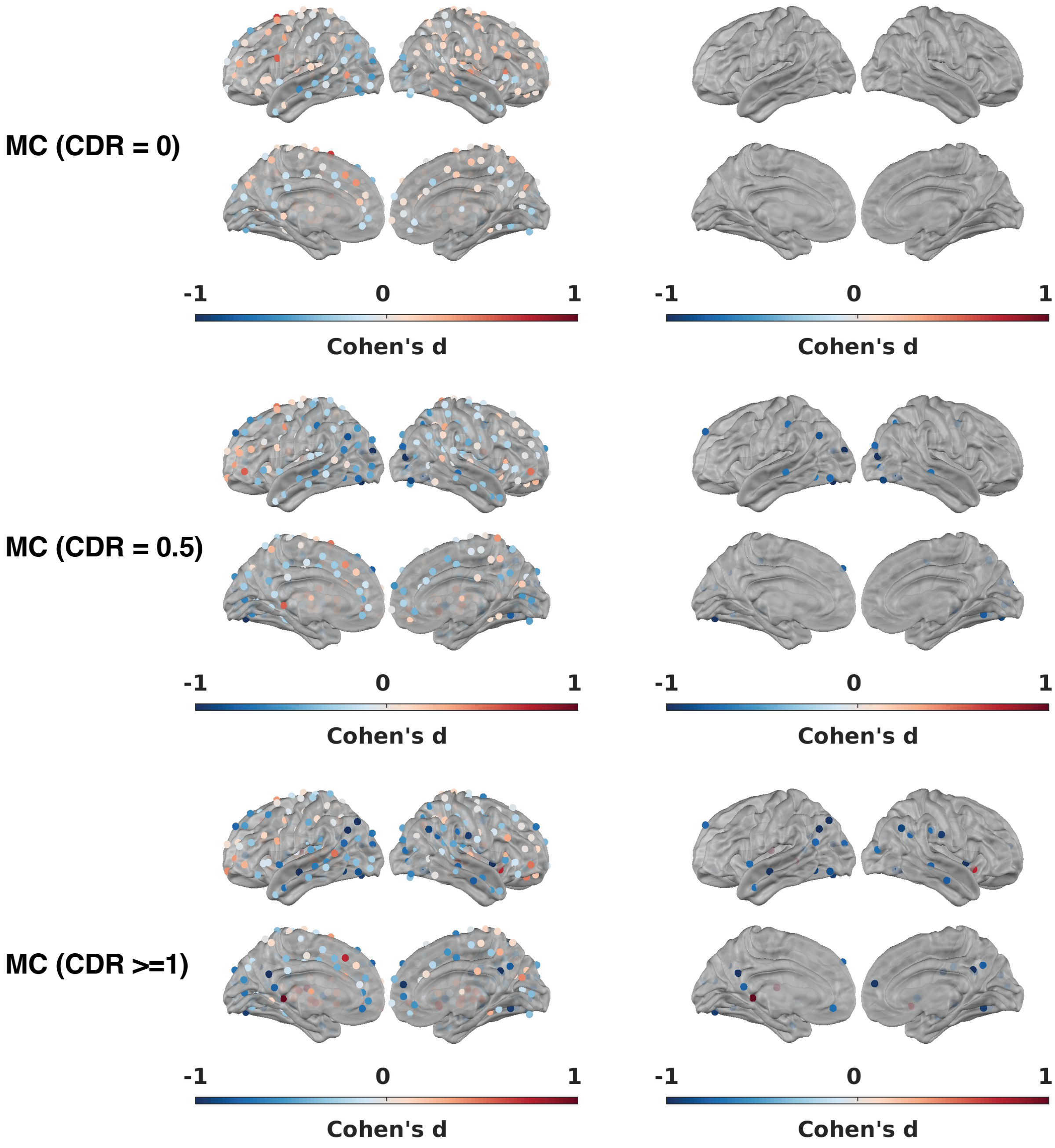

Supplementary Figure 7. Effect size of S difference from baseline and regions with a significant change from baseline (two-sample t-test, FDR < 0.05) for mutation carriers. Left column: Cohen's d for all ROIs. Right column: Cohen's d for ROIs passing an FDR < 0.05 threshold.

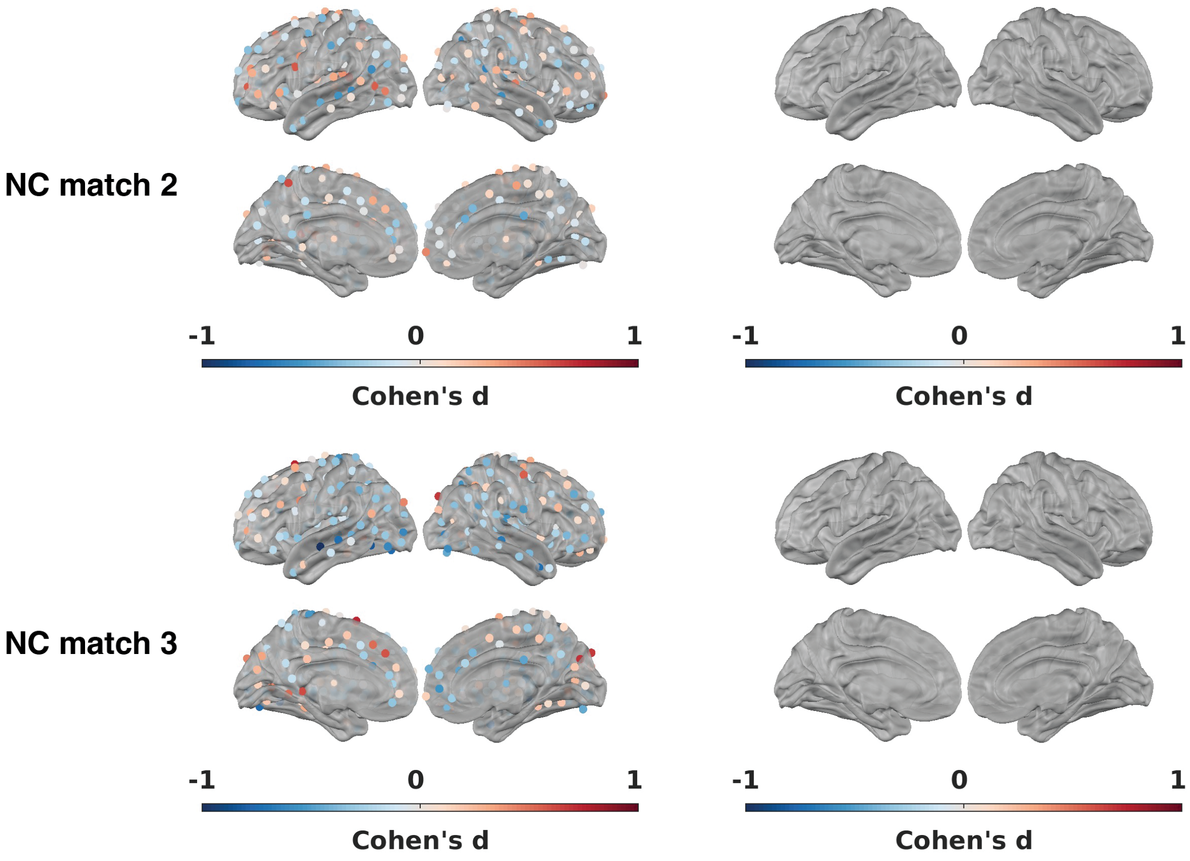

Supplementary Figure 8. Effect size of S difference from baseline and regions with a significant change from baseline (two-sample t-test, FDR-adjusted $p < 0.05$) for mutation non-carriers. Left column: Cohen's d for all ROIs. Right column: Cohen's d for ROIs passing an FDR-adjusted $p < 0.05$ threshold.

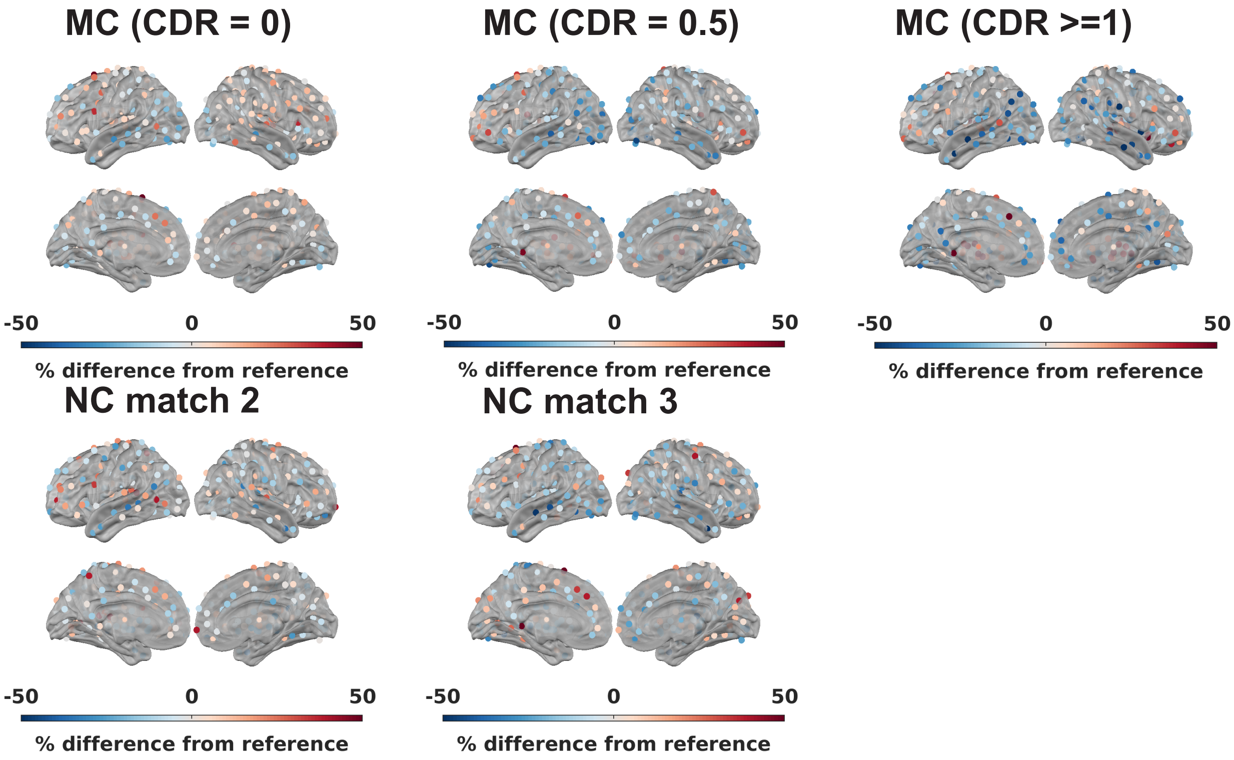

Supplementary Figure 9. Visualization of % strength change on the brain. Edge density = 5%.

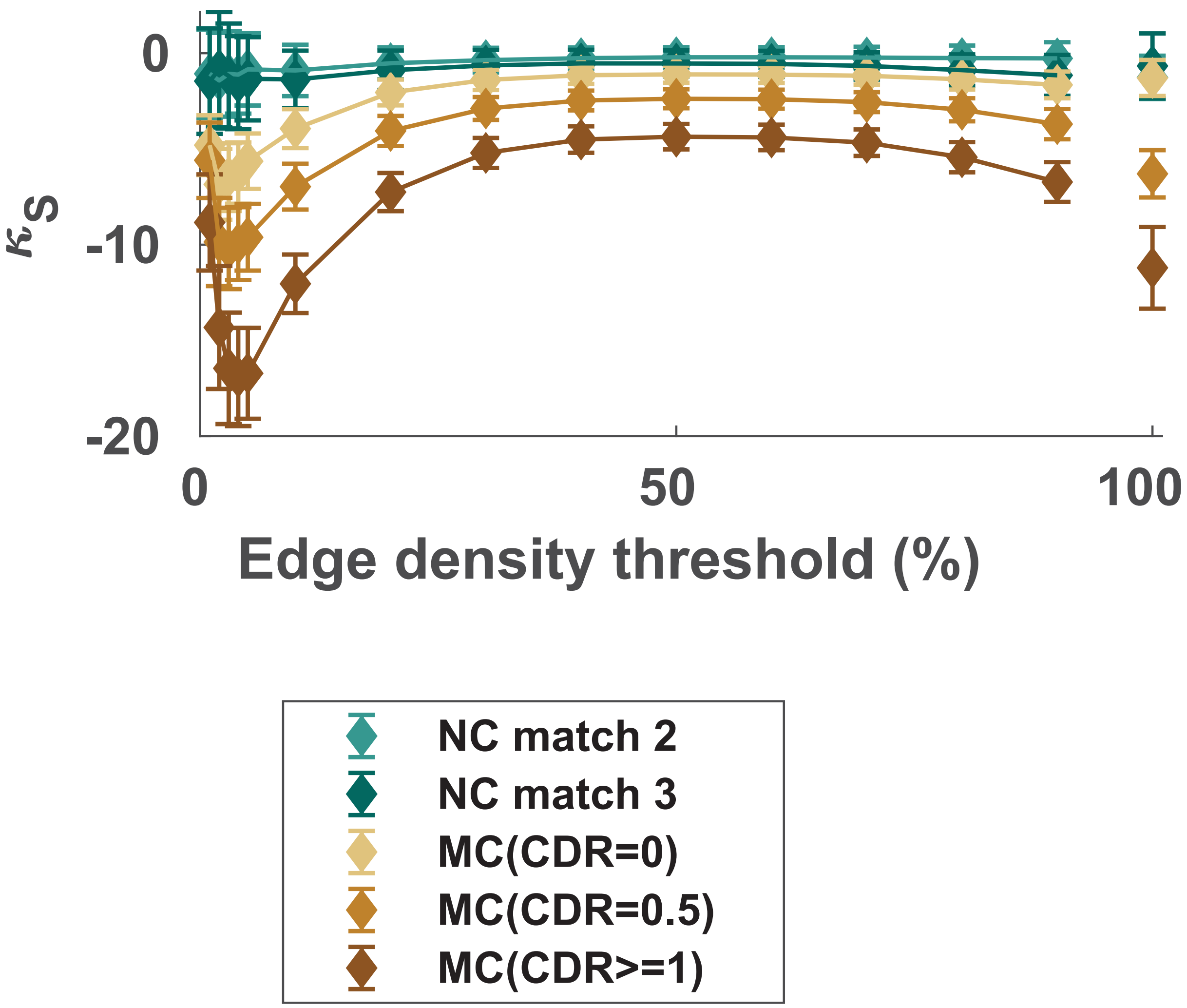

Supplementary Figure 10. Hub disruption in strength at different edge densities. Error bars indicate 95% confidence interval.

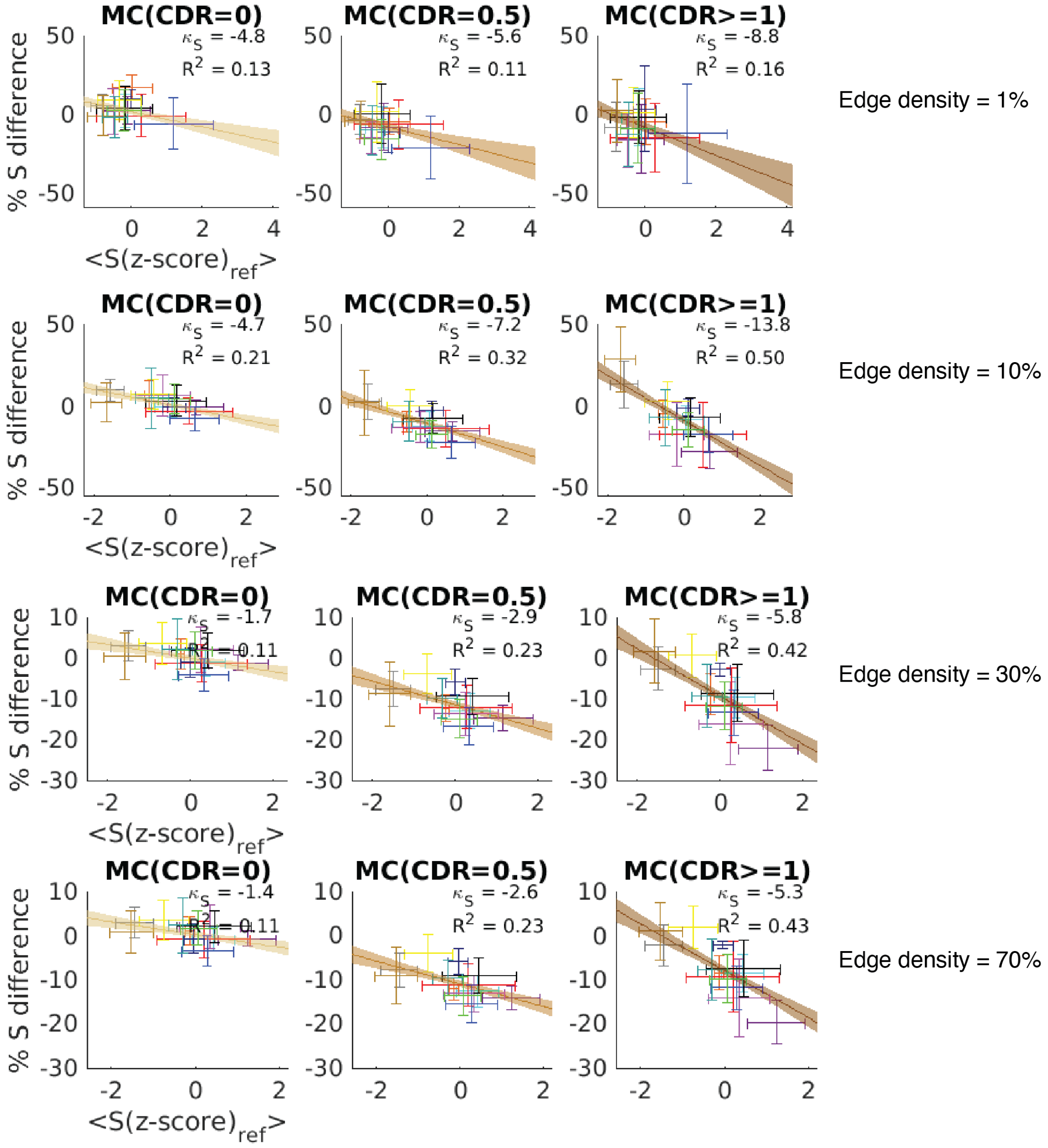

Supplementary Figure 11. Network summary of %S difference from reference against S(z-score) in NC match 1. Edge density = 1%, 10%, 30%, 70%. The cross hairs show the mean + standard deviation of the %S difference from reference against S(Z-score) for individual networks across all ROIs in the network. Shaded areas. The networks are color-coded according to Figure 1A. 13 Networks: SMd, somatomotor dorsal; SMl, somatomotor lateral; CO, cingulo-opercular; AUD, auditory; DMN, default mode network; Mem, memory network; Vis, visual network; FPN, frontoparietal network; SN, salience network; BG, basal ganglia; Thal, thalamus; VAN, ventral

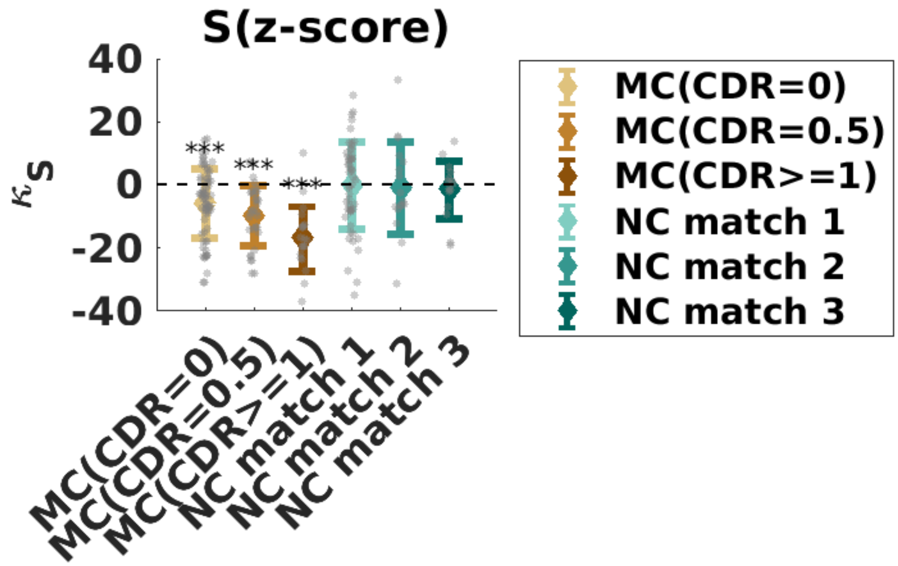

Supplementary Figure 12. Comparing the hub disruption indices with zero. The three mutation carrier groups have hub disruption indices with mean smaller than zero. The three non-carrier groups have hub disruption indices with mean not significantly different than zero. Error bars show the standard deviation. One-sample t-test with two-tails. Bonferroni-corrected p-values. * $p < 0.05$, ** $p < 0.01$, *** $p < 0.001$.

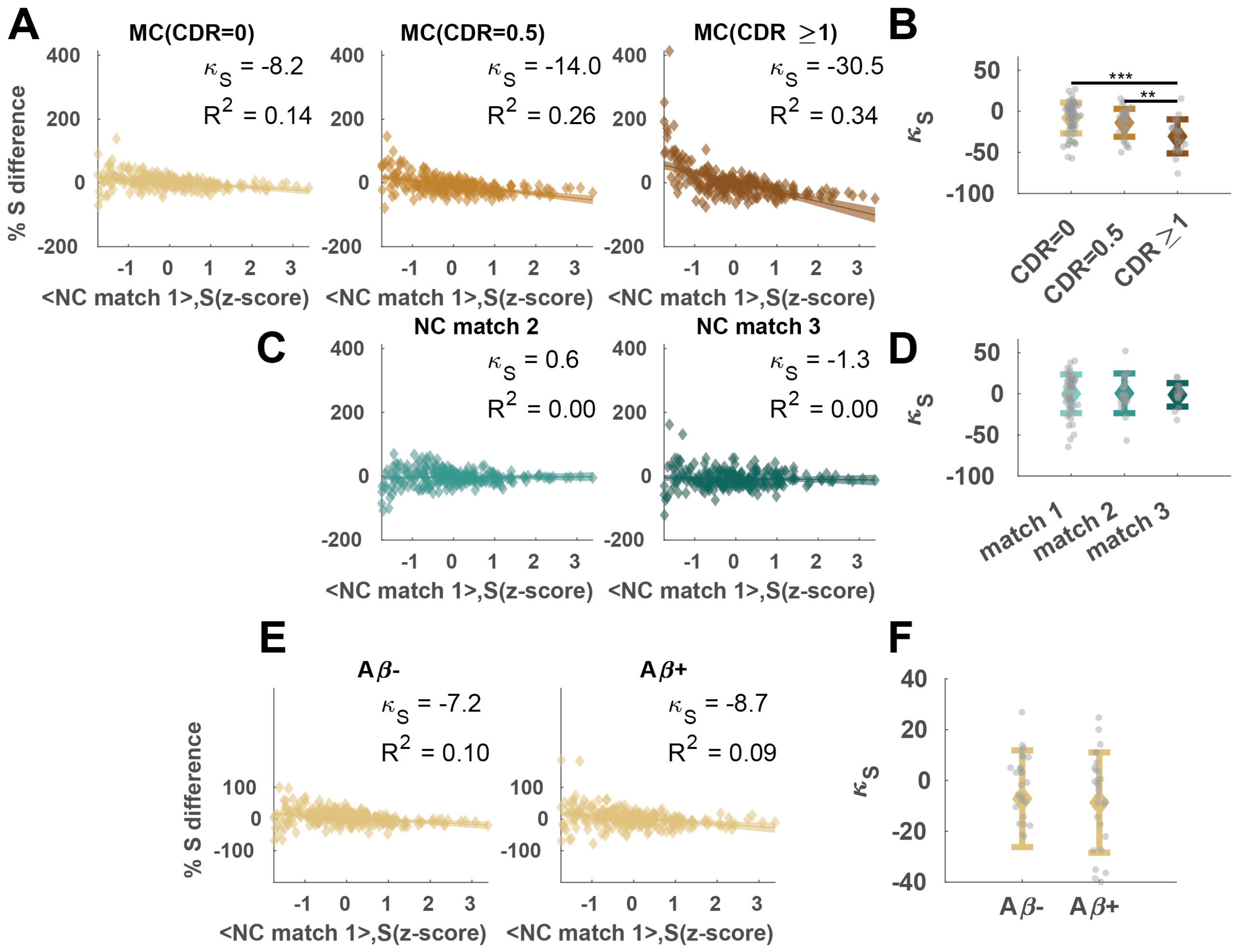

Supplementary Figure 13. Hub disruption across CDR stages (same analysis as Figure 3 but with gray matter volume regressed out from each ROI) (A) The % S difference against the reference S Z-score in MC groups. **(B)** Individual hub disruption index (κ_S) for MC groups. **(C)** The % S difference against the reference S Z-score in NC groups. **(D)** Individual κ_S for NC groups. **(E)** The % S difference against the reference S Z-score in subsets of Aβ- and Aβ+ participants in the MC(CDR=0) group. **(F)** Individual κ_S for Aβ- and Aβ+ participants in the MC(CDR=0) group. Shaded areas show 95% confidence interval. Error bars show mean and standard deviation. * $p < 0.05$, ** $p < 0.01$, *** $p < 0.001$. FDR-corrected.

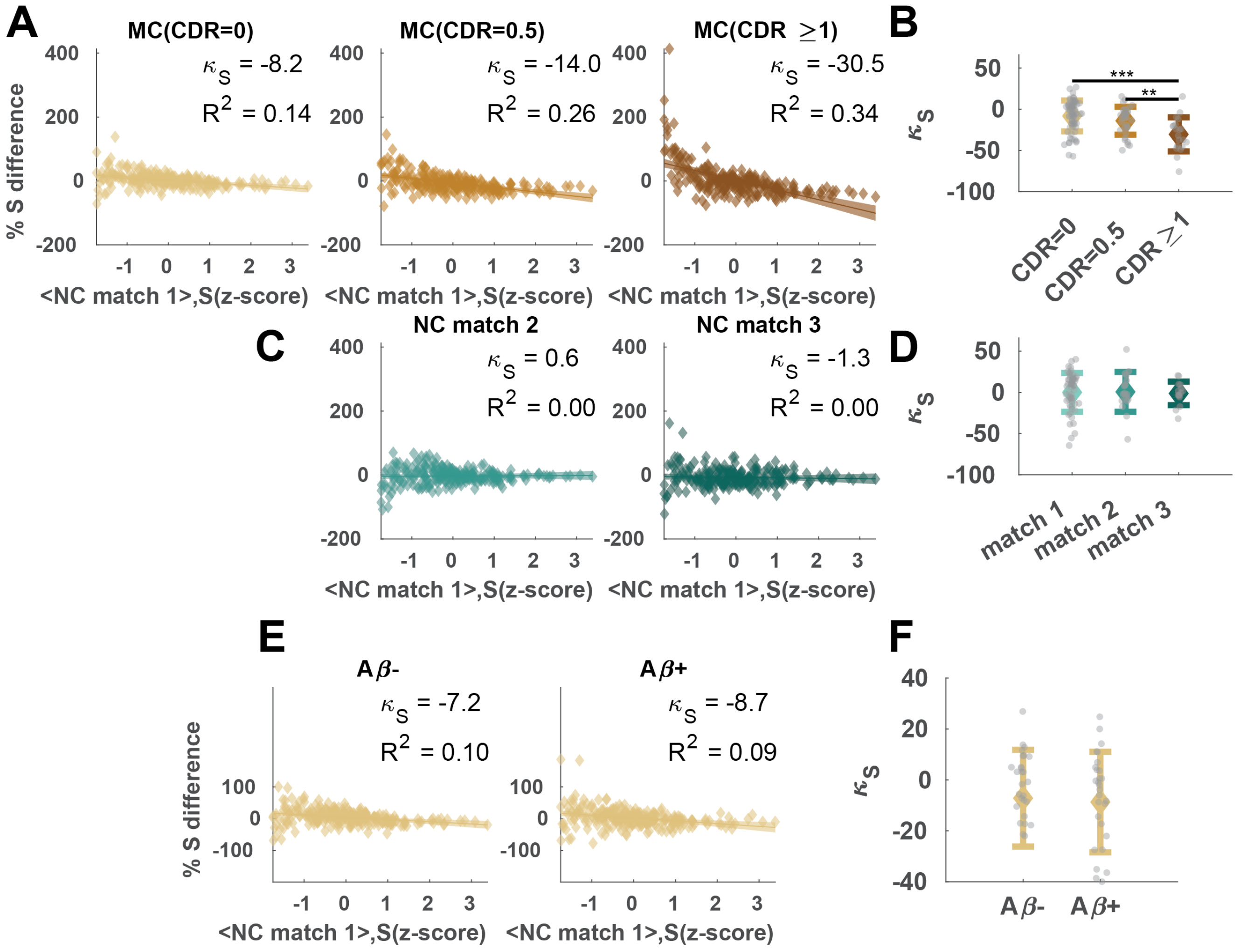

Supplementary Figure 14. Hub disruption across CDR stages at module centers versus connectors (same analysis as Figure 4 but with gray matter volume regressed out from each ROI) (A) The % S difference against the reference Pc Z-score for hub disruption calculation. **(B)** Individual hub disruption index (κ_S) for MC with respect to the group average Pc Z-score at NC match 1. **(C)** Individual hub disruption index (κ_S) for NC with respect to the group average Pc Z-score at NC match 1. **(D)** The % S difference against the reference Z for hub disruption calculation. **(E)** Individual hub disruption index (κ_S) for MC with respect to the group average Z at NC match 1. **(F)** Individual hub disruption index (κ_S) for NC with respect to the group average Z at NC match 1. Lines show linear fit and shaded areas indicate the 95% CI. * $p < 0.05$, ** $p < 0.01$, *** $p < 0.001$. FDR-corrected.

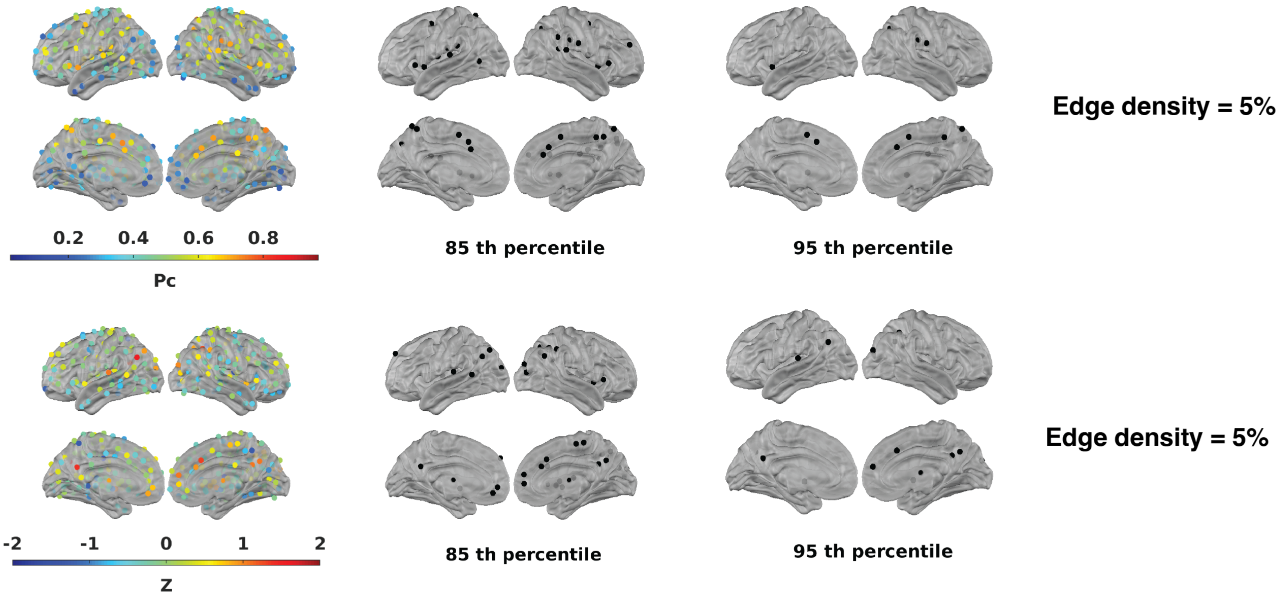

Supplementary Figure 15. Participation coefficient (Pc) and within-module strength Z-score (Z) and the areas passing 85th percentile or 95th percentile thresholds. Edge density = 5%.

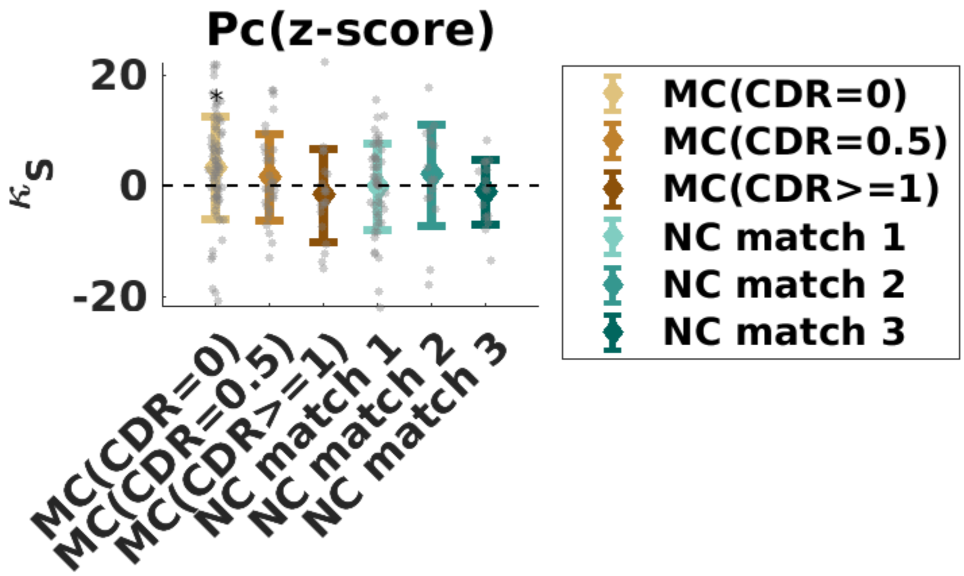

Supplementary Figure 16. Individual hub disruption indices (with Pc as reference) compared to zero. Error bars show the standard deviation. One-way ANOVA and post-hoc t-test with Bonferroni correction for six tests. * $p < 0.05$, ** $p < 0.01$, *** $p < 0.001$

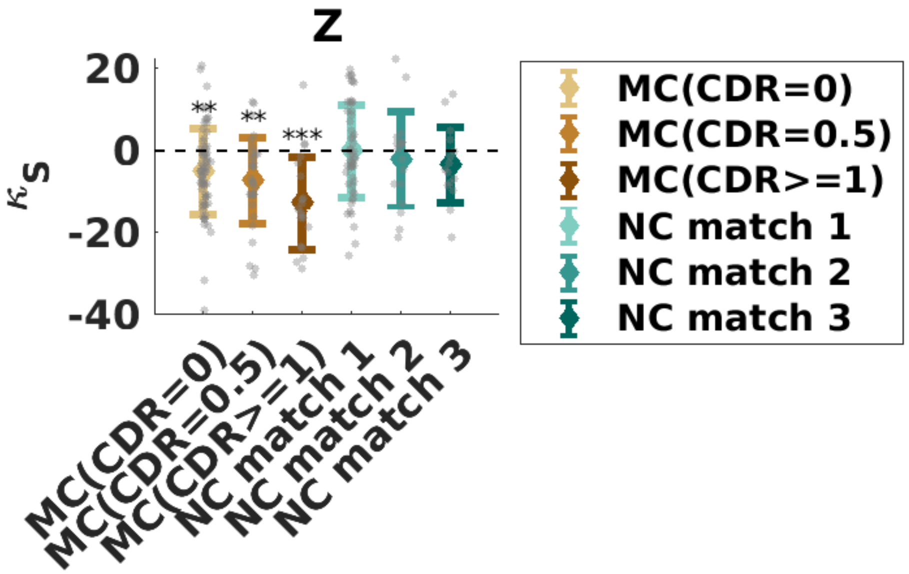

Supplementary Figure 17. Individual hub disruption indices (with Z as reference) compared to zero. Error bars show the standard deviation. One-way ANOVA and post-hoc t-test with Bonferroni correction for six tests. * $p < 0.05$, ** $p < 0.01$, *** $p < 0.001$

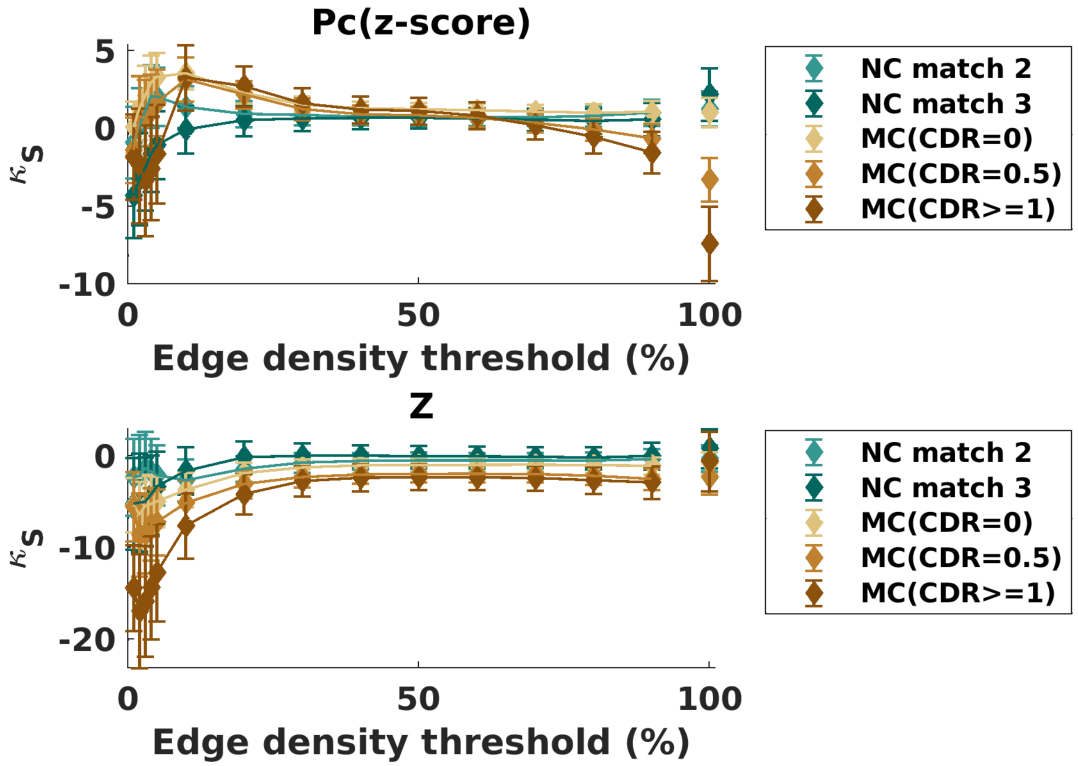

Supplementary Figure 18. Change in strength in relation to (top) participation coefficient (Pc) and (bottom) within-module strength Z-score (Z) across thresholds. Error bars show 95% CI.

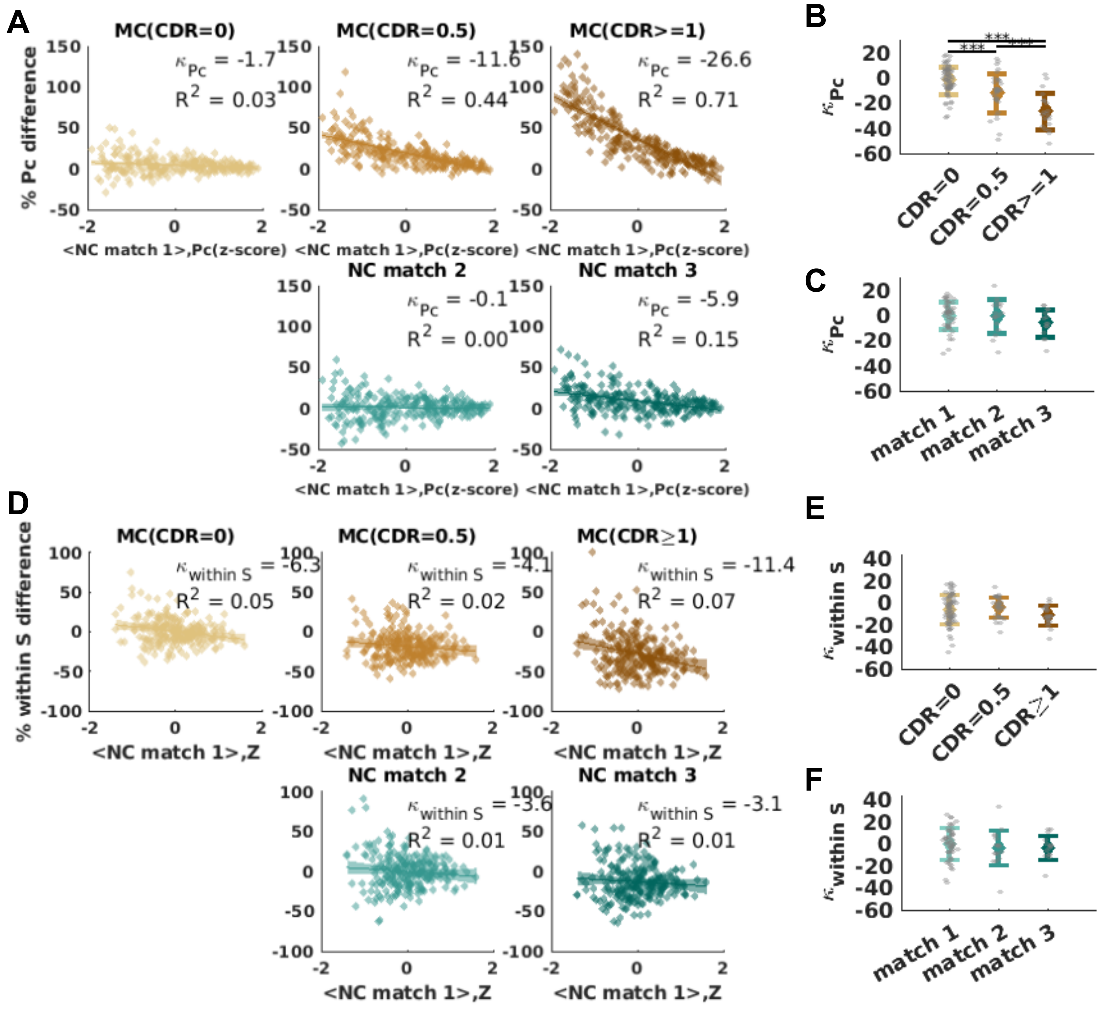

Supplementary Figure 19. Hub disruption across CDR stages at module centers versus connectors. (A) The % Pc difference against the reference Pc Z-score for hub disruption calculation. (B) Individual hub disruption index (κ_Pc) for MC with respect to the group average Pc Z-score at NC match 1. (C) Individual hub disruption index (κ_Pc) for NC with respect to the group average Pc Z-score at NC match 1. (D) The %within-module S difference against the reference Z for hub disruption calculation. (E) Individual hub disruption index ($\kappa\_withinS$) for MC with respect to the group average Z at NC match 1. (F) Individual hub disruption index ($\kappa\_withinS$) for NC with respect to the group average Z at NC match 1. Lines show linear fit and shaded areas indicate the 95% CI. * $p < 0.05$, ** $p < 0.01$, *** $p < 0.001$. FDR-corrected.

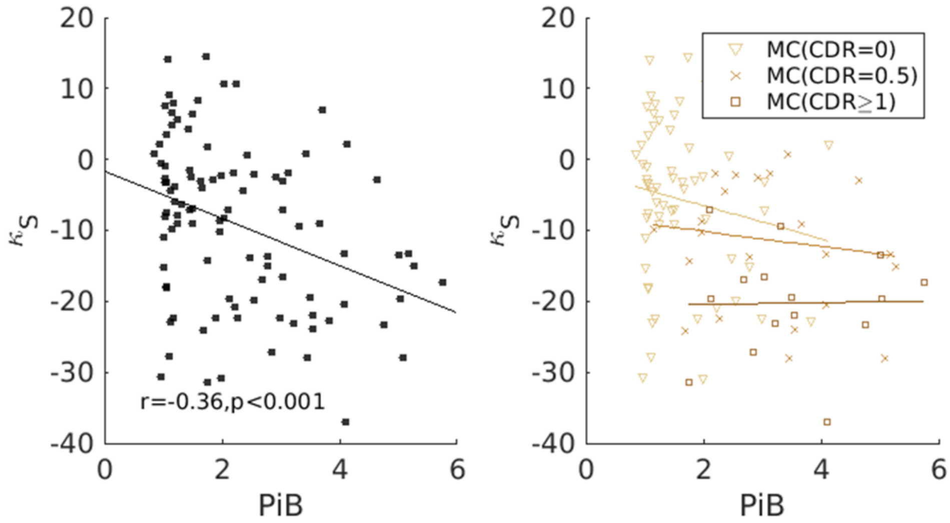

Supplementary Figure 20. PiB and κ_S correlation. (Left) Across all MCs. (Right) Separated into 3 MC groups.

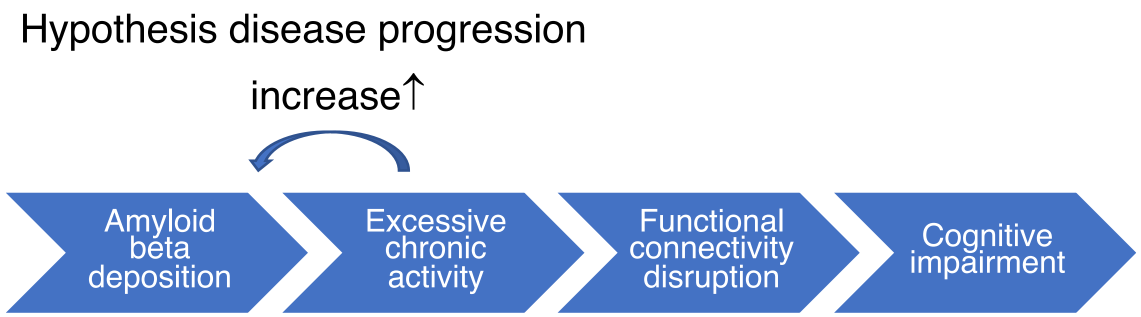

Supplementary Figure 21. Hypothesized disease progression model

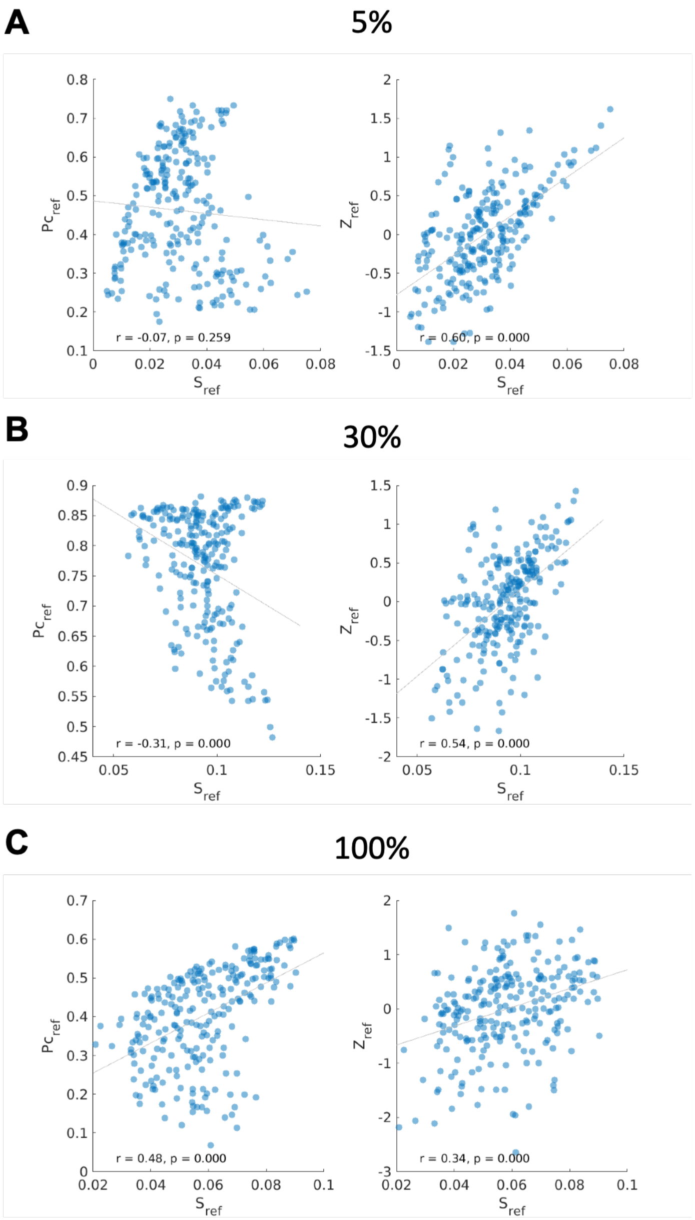

Supplementary Figure 22. **Correlation between Strength (S) and Participation Coefficient (Pc)/Within-module Strength Z-score (Z)**. A) at 5% edge density, B) at 30% edge density, C) at 100% edge density

References

Folstein, Marshal F., Susan E. Folstein, and Paul R. McHugh. 1975. "'Mini-Mental State.'" *Journal of Psychiatric Research* 12 (3): 189–98. https://doi.org/10.1016/0022-3956(75)90026-6.

Power, Jonathan D., Bradley L. Schlaggar, Christina N. Lessov-Schlaggar, and Steven E. Petersen. 2013. "Evidence for Hubs in Human Functional Brain Networks." *Neuron* 79 (4): 798–813. https://doi.org/10.1016/j.neuron.2013.07.035.

Wang, Guoqiao, Scott Berry, Chengjie Xiong, Jason Hassenstab, Melanie Quintana, Eric M. McDade, Paul Delmar, Matteo Vestrucci, Gopalan Sethuraman, and Randall J. Bateman. 2018. "A Novel Cognitive Disease Progression Model for Clinical Trials in Autosomal-Dominant Alzheimer's Disease." *Statistics in Medicine* 37 (21): 3047–55. https://doi.org/10.1002/sim.7811.

Wechsler, David. 1981. *WAIS-R Manual: Wechsler Adult Intelligence Scale-Revised*. Psychological Corporation.

———. 1987. *WMS-R: Wechsler Memory Scale--Revised : Manual*. Psychological Corporation.
